# IL-1β-induced modulation of gene expression profile in human dermal fibroblasts: the effects of Thai herbal Sahatsatara formula, piperine and gallic acid possessing antioxidant properties

**DOI:** 10.1186/s12906-016-1515-0

**Published:** 2017-01-10

**Authors:** Onusa Thamsermsang, Pravit Akarasereenont, Tawee Laohapand, Uraiwan Panich

**Affiliations:** 1 Department of Pharmacology, Faculty of Medicine Siriraj Hospital, Mahidol University, Bangkok, 10700 Thailand; 2 Center of Applied Thai Traditional Medicine, Faculty of Medicine Siriraj Hospital, Mahidol University, Bangkok, 10700 Thailand

**Keywords:** Thai herbal Sahatsatara formula, Thai herbal medicine, Inflammation, Antioxidant, Gene expression profile

## Abstract

**Background:**

Pain is the main symptom of most musculoskeletal disorders and can be caused by inflammation in association with oxidative stress. Thai herbal Sahatsatara formula (STF), a polyherbal formula, has been traditionally used for relieving muscle pain and limb numbness. This study aimed to investigate biologically active compounds of STF and its pharmacological effects related to antioxidant and anti-inflammatory activities.

**Methods:**

The identification of possibly active compounds of STF was performed by high performance liquid chromatography (HPLC). Moreover, this study also assessed the free radical scavenging activities of STF and its components using DPPH radical scavenging assay and their inhibitory effects on IL-1β-induced intracellular reactive oxygen species (ROS) formation in primary human dermal fibroblasts (NHDFs) using DCFDA-flow cytometry analysis. Modulation of human gene expression by STF and its active compounds was investigated by microarray analyzed through Gene Ontology (GO) classification and pathway enrichment analysis.

**Results:**

HPLC analysis has revealed the presence of gallic acid (GA) and piperine (PP) as the major compounds in STF extracts. Our finding discovered that STF and its active compounds (GA and PP) yielded free radical scavenging activities and abilities to inhibit IL-1β-induced cellular ROS formation in NHDFs. Furthermore, microarray analysis demonstrated that a total of 84 genes (54 upregulated and 30 downregulated) were significantly affected by IL-1β involved in inflammatory cytokines, chemokines, transcription factors, cell adhesion molecules and other immunomodulators participating in NF-κB signaling. The significantly upregulated genes in IL-1β-treated in NHDFs participate in interleukin and cholecystokinin (CCRK) signaling pathways. The GO analysis of the target genes showed that all test compounds including indomethacin, STF and its active compounds, can downregulate the genes involved in NF-кB signaling pathway in IL-1β-treated NHDFs compared to the cells treated with IL-1β alone.

**Conclusions:**

STF and its active compounds possessing antioxidant actions can modulate the effects of IL-1β-mediated alteration of gene expression profiles associated with inflammatory signaling in NHDFs.

**Electronic supplementary material:**

The online version of this article (doi:10.1186/s12906-016-1515-0) contains supplementary material, which is available to authorized users.

## Background

Pain is a non-specific symptom that can be found in several musculoskeletal disorders such as myofascial pain syndrome, fibromyalgia, tendinitis and arthritis [1]. Pathogenesis of pain is attributed to initiation of cellular damage and connective tissue damage through a number of mechanisms related to inflammation and oxidative stress. Some of secreted chemotactic molecules and mediators including cytokines can also trigger excessive reactive oxygen species (ROS) formation [2, 3].

IL-1β, a pro-inflammatory cytokines, plays a critical role during tissue injury and inflammation such as activating p38 mitogen-activated protein kinase (MAPK), c-Jun N-terminal kinase (JNK), nuclear factor-kappa B (NF-κB), extracellular signal-regulated kinase (ERK) signal transduction, increasing COX-2 activity and proinflammatory cytokines (IL-6, IL-8,TNF-α, IL-1) [4] and regulating ROS, collagen, proteoglycans synthesis in connective tissue [5]. While nonsteroidal anti-inflammatory drugs (NSAIDs) such as indomethacin, diclofenac, aspirin and ibuprofen are commonly used as anti-inflammatory and analgesic agents for relieving pain, the prolong use of NSAIDs could cause various side effects, such as gastrointestinal irritation, renal impairment, hepatotoxicity and allergic reaction [6].

Thai herbal Sahatsatara formula (STF), a polyherbal formula, recognized in the National List of Essential Medicines, is used to relief muscle pain, limb numbness and carminative. The formula is composed of 21 medicinal ingredients: camphor (1, 7, 7 – trimethylbicyclo (2.2.1) heptan −2- one), *Acorus calamus* L., *Atractylodes lancea* (Thunb.) DC., *Baliospermum solanifolium* (Burm.) Suresh., *Pistacia chinensis* subsp. *integerrima* (J. L. Stewart ex Brandis) Rech. f. Lagasca., *Picrorhiza kurroa* Royle ex Benth., *Plumbago indica* L., *Anethum graveolens* L*.,Cuminium cyminum* L., *Lepidium sativum* L., *Nigella sativa* L.,*Pimpinella anisum* L., *Clausena excavate* Burm.f., *Merremia vitifolia* (Burm.f.) Hallier.f, *Myristica fragrans* Houtt. (fruit and mace), *Piper nigrum* L., *Piper retrofractum* Vahl. *Terminalia chebula* Retz. (fruit and gall) and *Ferula assa-foetida* Regel*.* Previous studies demonstrated that STF showed effective anti-inflammatory activities by inhibiting COX-2 protein level and NO production by LPS-induced murine macrophages (RAW246.7). Moreover, STF and its component extracts also yielded the DPPH radical scavenging activity [7]. Recent clinical studies suggested that STF could attenuate symptoms from muscle pain [8] and osteoarthritis [9]. Although STF is widely used in Thai traditional practice, the evidence-based data regarding its active compounds and pharmacological effects which support the traditional usage are still unclear. This study thus aimed to; 1) identify active substances of STF by chromatography techniques, 2) determine the free radical scavenging properties of STF, its component extracts and its possibly active compounds using cell-free 2, 2-dipheny-1-picrylhydrazyl (DPPH) assay and 3) investigate antioxidant actions and the modulation by STF and its active compounds of the gene expression profile using microarray analysis using IL-1β-treated primary human dermal fibroblasts (NHDFs) culture model.

## Methods

### Materials and reagents

The STF composes of 21 components (Table 1). The crude powder of STF and its ingredients was supported by Center of Applied Thai Traditional Medicine, Faculty of Medicine Siriraj Hospital, Mahidol University. The characteristic of individual material was circumspectly authenticated by two experienced applied Thai traditional practitioners. The formula and ingredient powder was processed by the Manufacturing Unit of Herbal Medicines and Products Ayurved Siriraj under Good Manufacturing Practice (GMP) certification. Normal human dermal fibroblasts (NHDFs) and 2% fibroblast basal medium (FBM) were purchased from LONZA (LONZA, USA). The analytical grade ethanol and methanol reagents were purchased from Scharlau (Scharlau, Spain). All chemical reagents were obtained from Sigma-Aldrich (MO, USA).

**Table 1:** The percentage yields and IC_30_ values of free radical scavenging activities of STF extracts, its components and active compounds were obtained from DPPH assay and represented as Mean ± SEM. in triplicate experiments. IC_30_ values indicated concentrations of the test compounds required to scavenge DPPH radical by 30%

No.	Material name	Part of usage	Percentage of components in formula (W/W)^a^	% yields (W/W)^a^	DPPH assay IC_30_ value (μg/mL)
1	L-ascorbic acid	-	-	-	3.32 ± 0.22
2	Piperine	-	-	-	>120
3	Gallic acid	-	-	-	8.04 ± 0.94
4	STF formula	-	-	19.78	19.62 ± 1.13
5	1,7, 7 – trimethylbicyclo (2.2.1) heptan −2- one (analytical camphor)	crystal	1.40	-	>120
6	*Acorus calamus* L.	root	8.80	1.73	>120
7	*Anethum graveolens* L*.*	seed	1.00	2.82	>120
8	*Atractylodes lancea* (Thunb.) DC.	root	0.50	6.83	>120
9	*Baliospermum solanifolium* (Burm.) Suresh	root	8.00	0.74	90.86 ± 16.96
10	*Clausena excavate* Burm.f.	stem	4.80	2.30	98.47 ± 4.81
11	*Cuminium cyminum* L.	seed	0.80	6.26	75.67 ± 11.63
12	*Ferula assa-foetida Regel*	gum	1.00	27.89	>120
13	*Lepidium sativum* L.	seed	1.10	7.83	49.43 ± 5.42
14	*Merremia vitifolia* (Burm.f.) Hallier.f.	stem	0.80	1.49	25.17 ± 2.34
15	*Myristica fragrans* Houtt.	fruit	1.30	1.01	19.99 ± 1.17
16	*Myristica fragrans* Houtt.	mace	1.20	2.02	31.35 ± 3.13
17	*Nigella sativa* L.	seed	0.70	9.21	>120
18	*Pistacia chinensis subsp. integerrima (J. L. Stewart ex Brandis) Rech. f. Lagasca.*	root	0.60	11.09	65.57 ± 8.81
19	*Picrorhiza kurroa* Royle ex Benth.	root	0.40	7.70	84.96 ± 1.18
20	*Pimpinella anisum* L.	seed	0.90	5.40	78.54 ± 7.47
21	*Piper nigrum* L.	fruit	24.00	0.03	>120
22	*Piper retrofractum* Vahl.	fruit	9.60	6.03	>120
23	*Plumbago indica* L.	root	22.40	2.30	72.88 ± 6.84
24	*Terminalia chebula* Retz.	fruit	10.40	28.29	5.14 ± 0.52
25	*Terminalia chebula* Retz.	gall	0.30	8.83	2.35 ± 0.18

^a^(W/W) = gram unit

### Preparation of STF and its component extracts

The dried powder of STF and its ingredients were extracted 3 times by ethanol 80% at ratio of 1:10 (w/v) using ultrasonic sonicator machine (BANDELIN, Germany) controlled the ultrasonic frequency at 30 kHz and the temperature between 25 and 35 °C for 30 min and then filtrated through filter paper no. 4 (Whatman, England). The extraction solution was concentrated by rotary evaporator under 50 °C and at a pressure between 150 and 180 mbar (Buchi, Switzerland) and each sample was stored at −80 °C prior to lyophilization. The calculated % yield (w/w) was shown in Table 1. The lyophilized powder (10 mg) was reconstituted with 80% ethanol, vortexed and centrifuged at 22136.4 g for 10 min at 4 °C. The extraction solution was then filtered through 0.22 μm polyvinylidene difluoride membrane (PVDF) (Vertical Chromatography, Thailand) to remove the extraction residues.

### Identification of active compounds by HPLC analysis

**Fig. 1:**
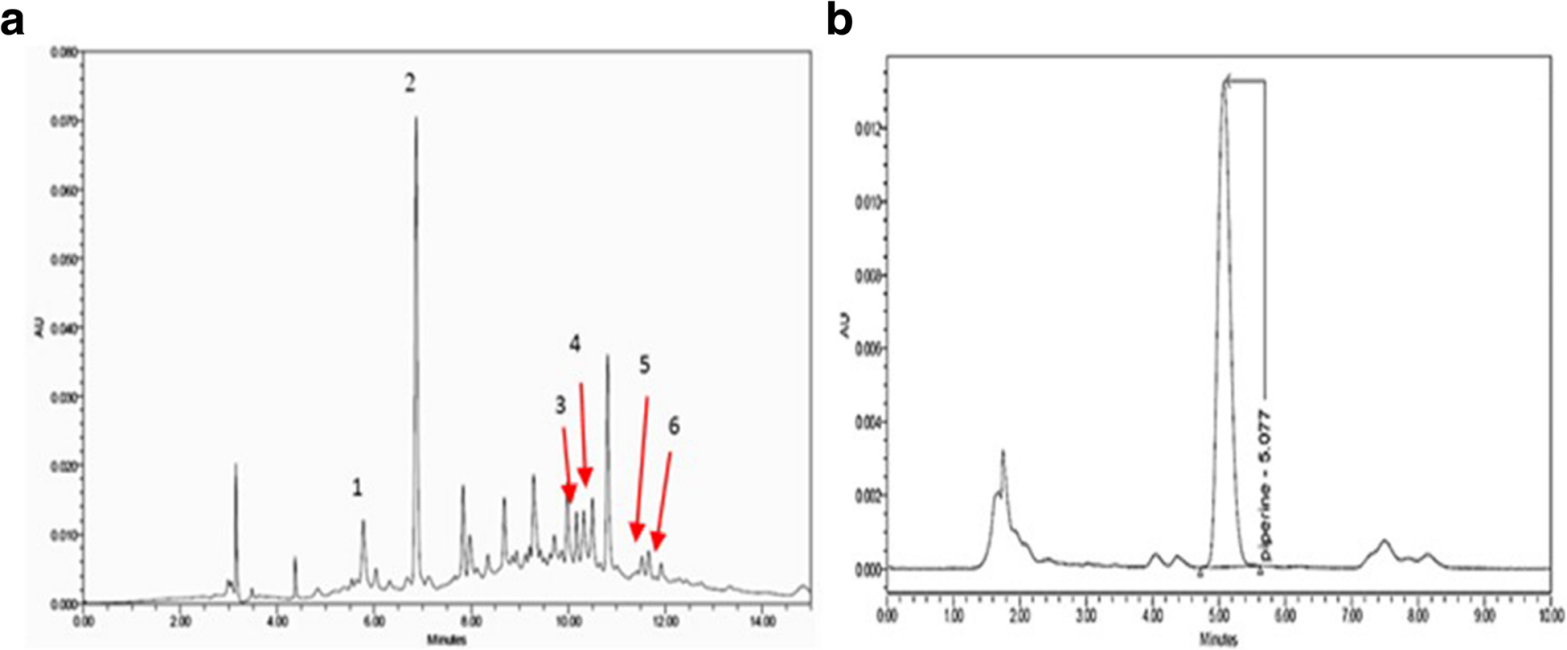
The HPLC fingerprints of phenolic compounds (**a**) and piperine (PP) (**b**) in STF extracts. Peak details; 1: kojic acid (RT: 6.103 min); 2: gallic acid (GA) (RT: 6.940 min); 3: caffeic acid (RT: 10.211 min); 4: vanellic acid (RT: 10.433 min); 5: p-coumaric acid (RT: 11.524 min); 6: ferulic acid (RT: 12.039 min)

HPLC analysis was applied for identification and quantification of the STF extracts and its possible active compounds. Briefly, phenolic compounds (including gallic acid (GA), p-coumaric acid, kojic acid, caffeic acid, ferulic acid, and vanillic acid) and piperine (PP) were selected as reference markers from literature-based review of STF components. Chromatographic profiles of STF extract and reference compounds were detected by the Waters 2998 Photodiode Array (PDA) and the analytical procedure was performed using Alliance e2695 (Water, USA) and Waters Empower 2 software. Phenumenix C18 5 μm (4.6 mm × 250 mm) and Sunfire C18 5 μm (4.6 mm × 150 mm) (Water, Ireland) were used as a stationary phase for analysis of phenolic compounds and piperine, respectively. The mobile phases at a flow rate 1 mL/min composed of 1% acetic acid in Milli-Q water or 10 mM ammonium acetate (pH 6.8) (solvent A) for phenolics and PP, respectively, and 100% methanol (solvent B) for both phenolics and PP. For analysis of phenolic substances, a gradient condition was used as follows: 0 min, 98% A/2% B; 3 min, 90% A/10% B; 5 min, 80% A/20% B; 7 min, 70% A/30% B; 13 min, 85% A/15% B; 14 min, 100% B; 16–18 min, 98% A/2% B. The peaks were detected at wavelengths 210–400 nm. For analysis of PP, an isocratic elution with 25% A/75% B was used and chromatograms were detected at 340 nm. The identification of phenolic compounds and PP in STF extracts was presented in Fig. 1a and b. The GA and PP contents in STF were 0.175 (W/W) % and 0.602 (W/W) %, respectively.

### Screening of free radical scavenging activity

2, 2-dipheny-1-picrylhydrazyl (DPPH), a stable free radical molecule, was commonly used to determine free radical scavenging activity as previously described [10]. STF, its component extracts and reference compounds (GA, PP and L-ascorbic acid) were prepared by diluting serially with 80% ethanol (3.75, 15, 30, 60, 120 μg/mL). The absorbance was detected spectrophotometrically at 520 nm at 0 and 15 min by a microplate reader SpectraMAX M5 (Molecular Devices, CA).

### Cell culture and treatments

NHDFs divided from adult female skin were cultured in 2% FBM supplemented with 1% penicillin (100 units/mL) and streptomycin (100 μg/mL) under 37 °C, 5% CO_2_ in incubator. Upon reaching 80–100% confluency in T75 flask, cells were treated with indomethacin, STF extracts and its reference markers (GA and PP). Stock solutions of all test compounds were freshly prepared with 80% ethanol and filtrated through 0.22 μm PVDF filter. To induce inflammation and oxidative stress of NHDFs, IL-1β was used in this study and diluted with serum-free medium and indomethacin applied for a positive control was dissolved in 1.80% dimethyl sulfoxide (DMSO) solution.

### Cell viability assay

MTT assay was performed to determine non-toxic concentrations of vehicles (80% ETOH and DMSO) and test compounds for further cellular studies. Briefly, cells were pre-incubated with vehicle or test compounds at vary concentrations for 24 h and 200 μL of MTT reagent (0.2 mg/mL) was then added into each well and incubated for 4 h. To dissolve formazan product, 200 μL of DMSO reagent were added into each well followed by measuring the absorbance at 595 nm using a spectrophotometer (SpectraMAX M5, Molecular Devices, CA).

### Determination of intracellular ROS formation

Intracellular ROS formation in NHDFs was determined by flow cytometry using 2′, 7′-dichlorofluorescein diacetate (DCFH-DA), a stable and non-fluorescent dye. To investigate ROS production induced by IL-1β, NHDFs were treated with IL-1β (0.1, 0.5, 1, 10 ng/mL) for 30 min. To assess inhibitory effects of STF extracts and its active compounds on ROS generation. NHDFs were pre-treated with GA (0.3, 1, 3 μg/mL), PP (1, 3, 10, 30 μg/mL), STF extracts (3, 10, 30 μg/mL) and vehicle reagent (ETOH 80%) following treated concentrations for 30 min prior to treatment with IL-1β (1 ng/mL) for 30 min and then incubated with 5 μM DCFH-DA for 30 min. The oxidized DCFDA indicating ROS formation was measured using flow cytometry (BD Biosciences, USA) and fluorescence intensity was analyzed by FlowJo vX.0.7 software (TreeStar Inc, Ashland, OR, USA).

### Gene expression profiling study

To assess the effect of STF and its active compounds on human-whole genome expression. NHDFs were treated with indomethacin (50 μM), GA (0.3, 1, 3 μg/mL), PP (1, 30 μg/mL) and STF extracts (3, 10, 30 μg/mL) for 30 min prior to treatment with IL-1β (1 ng/mL) for 4 h. Briefly, total mRNA of each treatment was isolated by Illutra RNA spin Mini RNA isolation Kit (GE Healthcare, UK). The concentration and purity of isolated mRNA were determined using NanoVue UV/visible spectrophotometer (GE Healthcare, UK) and Qubit® quantitation assay kit (Life Technologies, USA) followed by the instructions. To estimate RNA integrity, RNA samples were analyzed using Agilent RNA 6000 Nano kit (Agilent Technologies, USA) with Agilent 2100 Bioanalyzer (Agilent Technologies, USA). The 28S/18S ratio of total RNA was equal and greater than 2.0 and the RNA Integrity Number (RIN) was greater than 7.0. Then, the total mRNA was converted into complementary RNA (cRNA), amplification and biotin labelling were carried out according to the Illumina TotalPrep™ RNA Amplification kit (Life Technologies, USA). Biotin-labelled cRNA (750 ng) was hybridized with Human HT-12 V4 BeadChip (Illumina, USA) following the whole genome expression direct hybridization assay protocol (Illumina, USA). Briefly, 15 μL of each cRNA sample was pipetted onto the HumanHT-12 v4 BeadChip (Illumina, USA) and chamber was then incubated in hybridization oven at 58 °C for 14–20 h. The BeadChip was washed out and then stained with Cy3-STFeptavidin (GE Healthcare, UK), which was further scanned with HiScan array scanner (Illumina, USA). The backgrounds of intensity were subtracted and operated under GenomeStudio™ gene expression module 1.0 software. All data were exported as Binary Manifest Files (*.bgx), then converted to a Comma-Separated Values (CSV) file imported into Microsoft Excel program for Window.

### Microarray data analysis

The signal intensities of samples were normalized and analyzed using Microsoft Excel program version 2013. Data analysis was performed as described in Additional file 1: Figure S1. Twelve human housekeeping genes (Additional file 1: Table S1) were applied for normalization of each gene intensity using the normalization factor to adjust the unequal distribution of signal intensity. The normalized and non- normalized microarray data had deposited in the Gene Expression Omnibus (GEO) databases with accession number GSE86798 (https://www.ncbi.nlm.nih.gov/geo/query/acc.cgi?acc=GSE86798). The normalized values were used to calculate fold change, which was converted into logarithmic base 2. To determine the up-regulated or down-regulated genes, the Student’s *t*-test was performed to estimate significant difference of gene expression based on *P* < 0.05 with fold change that cut off ≥ 2 or ≤ 0.5 times compared with untreated control group or IL-1β-treated group without compound treatment. To investigate the effects of STF extract and its active compounds on gene expression analyzed by Gene Ontology (GO) classification and pathway enrichment analysis using PANTHER web-database followed a protocol [11].

### Quantitative real-time polymerase chain reaction (qRT-PCR)

qRT-PCR was used to validate differential gene expression from IL-1β stimulation. Total RNA of all samples were isolated by Illutra RNA spin Mini RNA isolation Kit (GE Healthcare, UK) and converted into complementary DNA (cDNA) using ImProm-II ™ reverse transcription system (Promega, USA). The primers were designed and verified by Primer-Blast from NCBI website (https://www.ncbi.nlm.nih.gov/tools/primer-blast/). The PCR reaction was performed using ABI Prism 7300 Real Time PCR System (Applied Biosystems, USA) and KAPA SYBR Fast qPCR kits (Kapa Biosystems, UK). The fast amplification condition: enzyme activation step at 95 °C for 3 min, annealing step at 60 °C for 30 s, and an extension step at 72 °C for 3 s for a total of 40 cycles. The list of primer sequences were described in (Additional file 2: Table S1). The relative normalized genes expression were estimated using ΔΔCt method and each gene expression result was normalized with GADPH being housekeeping gene and internal control in the experiment. The experiment was carried out in triplicate. The results of qRT-PCR analysis were shown in Additional file 2: Figures S1 and S2.

### Statistical analysis

Data were expressed as means and standard deviation (SD) or standard error of the mean (SEM). The significantly statistical values concerned at *P* <0.05 were estimated by Unpaired t- test and one-way analysis of variance (ANOVA) followed by Dunnett’s post hoc test using GraphPad Prism version 5 for Windows (GraphPad Software Inc., San Diego, CA, USA).

## Results

### The antioxidant activities of STF and its ingredients

The free radical scavenging activities of STF extracts, its components and possible active compounds including GA and PP were examined by DPPH assay. As shown in Table 1, STF extracts, GA and L-ascorbic acid had abilities to scavenge DPPH radical in a dose-dependent manner, whereas PP had no free radical scavenging properties. Based on IC_30_ value, STF’s components which ranked in the top five for free radical scavenging activities were *Terminalia chebula* Retz. (gall), *Terminalia chebula* Retz. (fruit), *Myristica fragrans* Houtt. (fruit), *Merremia vitifolia* (Burm.f.) Hallier.f. and *Myristica fragrans* Houtt. (mace), respectively. In addition, the extracts from analytical camphor and gum of *Ferula assa-foetida* Regel showed no free radical scavenging properties.

### Cytotoxicity assessments: inflammatory inducer, solubilizing vehicles, STF and its active compounds

**Fig. 2:**
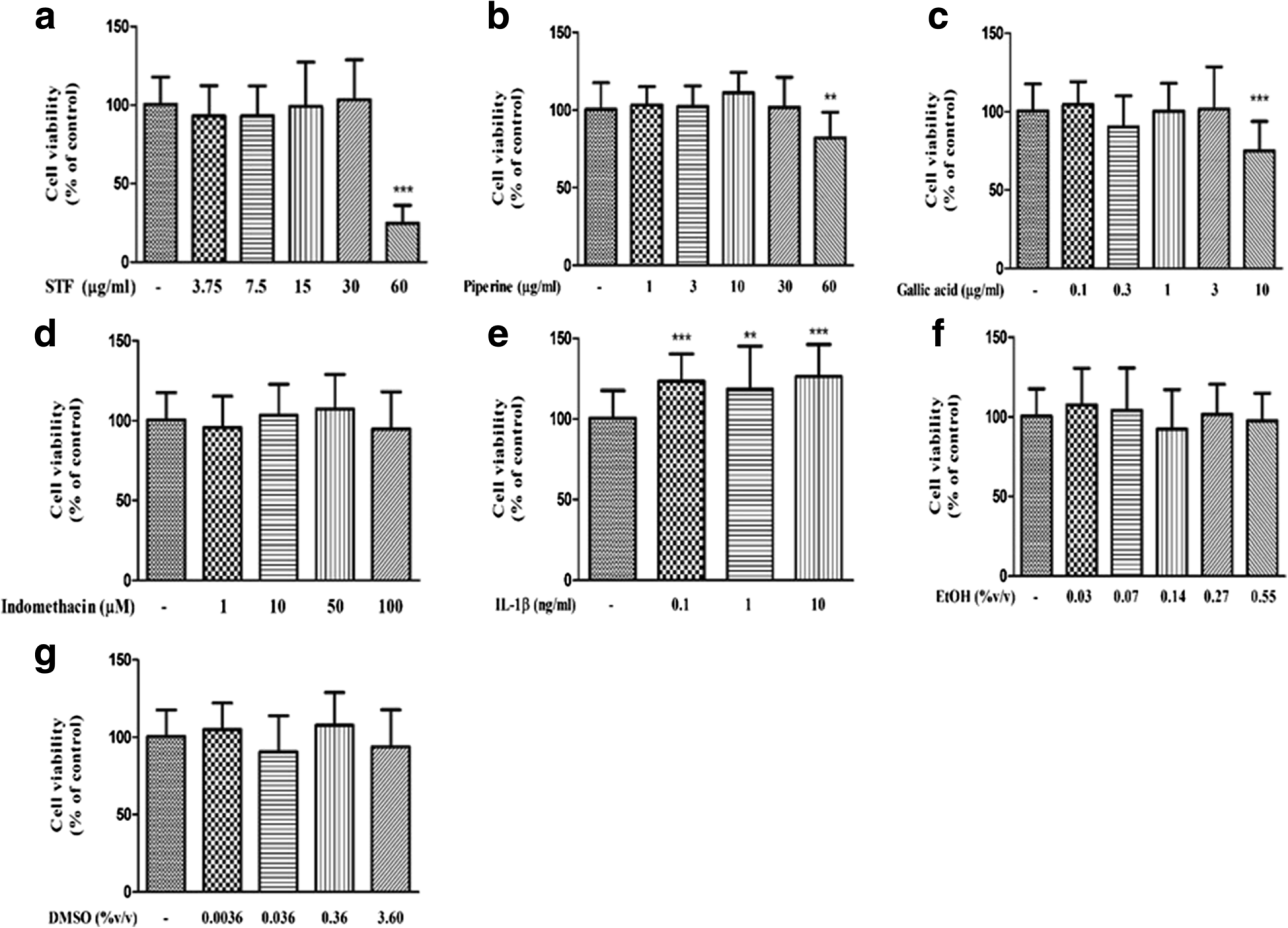
Cytotoxicity of STF extracts (**a**), Piperine (PP) (**b**), Gallic acid (GA) (**c**), Indomethacin (**d**), IL-1β (**e**), ETOH80% (**f**) and DMSO (**g**) on NHDFs viability. The results were obtained from at least triplicate experiments, One-Way ANOVA (Dunnett’s post hoc test) was used to determine statistical significance and the data are represented as Mean ± SD. *P <0.05; **P <0.01; ***P <0.001 compared with control group

The results showed that treatment of NHDFs with vehicles and test compounds including STF extracts, GA and PP (3.75 to 30 μg/mL), indomethacin (1–100 μM) and IL-1β (0.1,1,10 ng/mL) for 24 h did not affect NHDFs viability compared to untreated control cells (Fig. 2).

### The inhibitory effects of STF extracts and its active substances on intracellular ROS formation induced by IL-1β in NHDFs

**Fig. 3:**
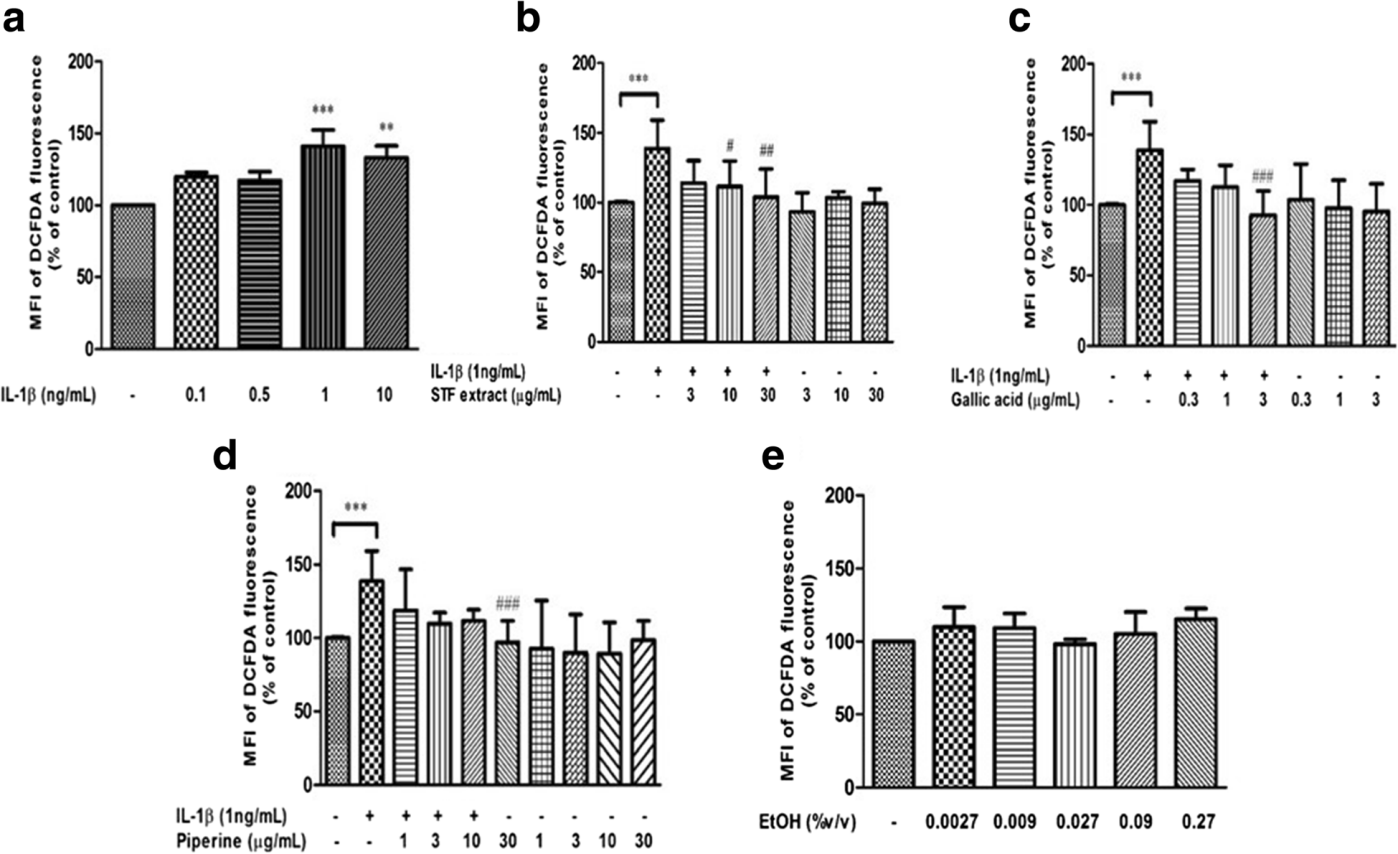
The effects of STF extracts and its active compounds on ROS formation was assessed by DCFDA-flow cytometry. IL-1β significantly enhanced ROS formation in a dose-dependent manner in IL-1β-treated NHDFs (**a**). The pretreatment of STF extracts (**b**), GA (**c**) and PP (**d**) can attenuate IL-1β-induced intracellular ROS levels whereas, treatment with each compound alone (**b**-**d**) and vehicle reagents (ETOH 80%) (**e**) didn’t affect ROS generation. DCFDA intensity data were presented as Mean ± SD at least three experiments. The statistical significant between control and IL-1β-treated cells was evaluated by Unpaired *t*-test and between IL-1β-treated and test compound–treated cells was assessed by one-way ANOVA followed by Dunnett’s post hoc test. **P < 0.01, *** *P* < 0.001 compared with control. ^#^ *P* < 0.05, ^##^ *P* < 0.01 and ^###^ *P* < 0.001 compared with IL-1β-treated cells

As shown in Fig. 3a, IL-1β (1 ng/mL and 10 ng/mL) significantly enhanced ROS generation in NHDFs (*P* <0.01 and <0.001) determined by flow cytometry. The results of oxidized-DCFDA fluorescence indicated that STF extracts (10 and 30 μg/mL) and the highest dose of GA (3 μg/mL) and PP (30 μg/mL) significantly suppressed ROS production in NHDFs compared with IL-1β-induced cells (Fig. 3b-d). Additionally, treatment with vehicle control (Fig. 3e), STF extracts or its active compounds alone did not affect ROS formation in NHDFs.

### IL-1β modulated gene expression profile in fibroblasts: the effects of STF and its active compounds

**Fig. 4:**
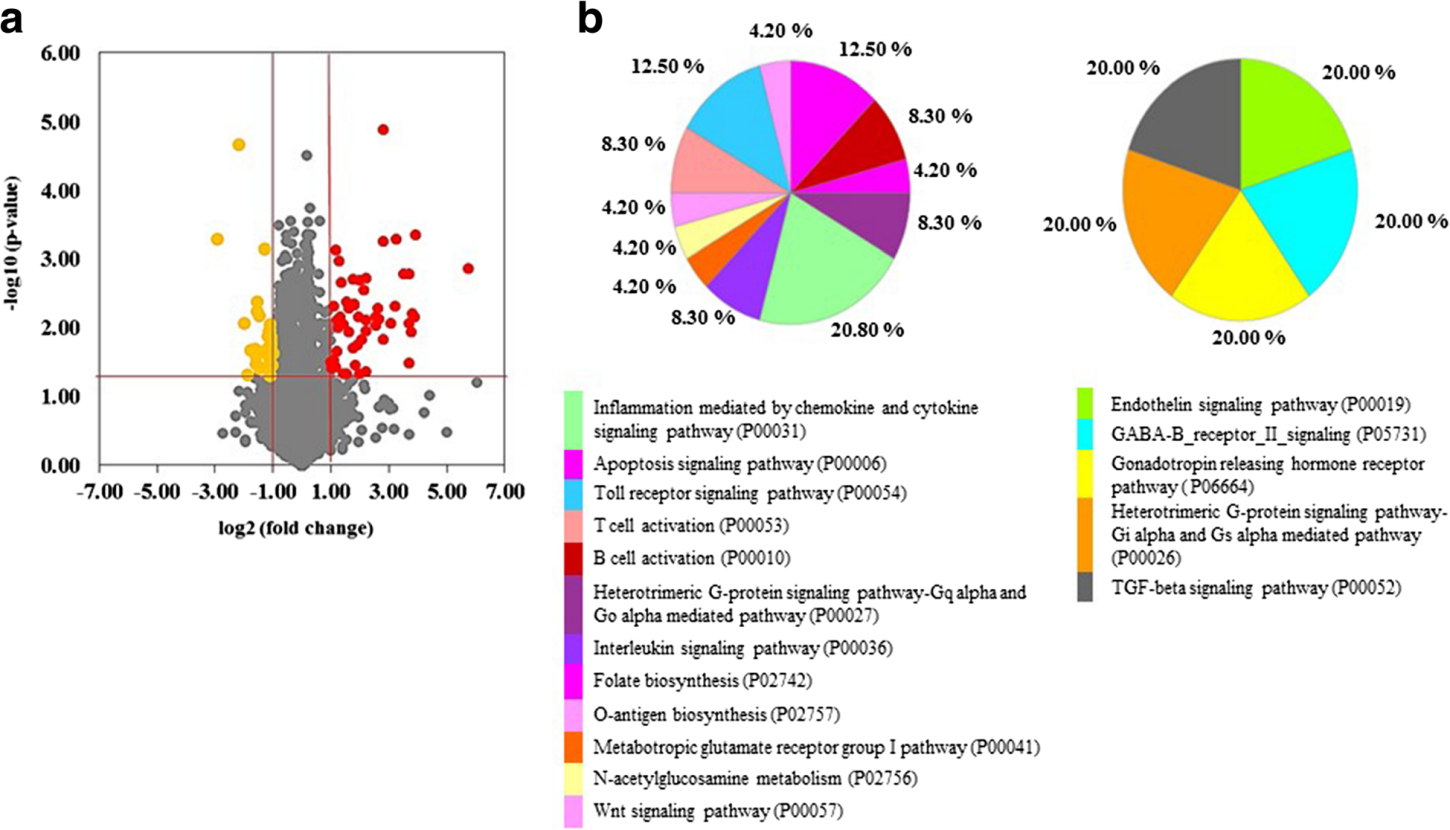
The differential expression of genes in NHDFs treated with IL-1β when compared with non-treated cells. The significantly altered genes (30 down-regulated and 54 up-regulated) induced by IL-1β were indicated by yellow dots and red dots, respectively (**a**). The Gene Ontology (GO) classification of 84 altered genes was performed using PANTHER classification system analysis (**b**).

To investigate the role of IL-1β on transcriptional profiling of fibroblasts, the volcano plot of whole human gene expressions (47,231 probes) demonstrated the differential expression of IL-1β-treated cells compared with untreated cells. Following the analytical criteria, the volcano plot was shown total of 84 genes with significantly up-regulated expression (54 genes) and down-regulated expression (30 genes) detected in IL-1β-treated cells compared to untreated control cells (Fig. 4a and Additional file 3: Table S1 and S2). The gene GO analysis of up- and down- regulated genes was shown in Fig. 4b. The up-regulated genes in IL-1β-treated cells mainly involved signaling pathways such as inflammation, apoptosis, heterotrimeric G-protein activation, and adaptive immune activation. However, only two pathways from PANTHER pathway enrichment analysis comprising BICR3, CXCL1, CXCL2, NFKBIA, IL-8 and IL-6 were significantly moderated by IL-1β (Table 3). 22 genes of the 54 up-regulated genes were targeted genes associated with NF-кB transcription, including related transcription factors, chemokines, cytokines and immunoreceptors regulated by NF-кB (Table 2).

**Table 2:** The summery of 22 target genes involved in transactivation of NF-кB induced by IL-1β in NHDFs. All gene description and functional annotation were referred from PANTHER and NCBI databases

Group	Gene symbol	Description/Function
Transcription factor and their modulators	CEBPD	CCAAT/Enhancer Binding Protein (C/EBP), Delta /regulation of genes involved in immune and inflammatory responses (macrophage)
	IRF1	Interferon Regulatory Factor 1/ an activator of interferons alpha and beta transcription, regulations of apoptosis and tumor-suppression
	NFKB1	Nuclear Factor Of Kappa Light Polypeptide Gene Enhancer In B-Cells 1/ sequence-specific DNA binding transcription factor activity and transcription factor binding
	NFKBIA (IκBα)	Nuclear Factor Of Kappa Light Polypeptide Gene Enhancer In B-Cells Inhibitor, Alpha / inhibits the activity of dimeric NF-kappa-B/REL complexes
	NFKBIZ (IκBZ)	Nuclear Factor Of Kappa Light Polypeptide Gene Enhancer In B-Cells Inhibitor, Zeta / a member of the ankyrin-repeat family, produce IL6 secretion induced by lipopolysaccharide.
	TNFAIP3	Tumor Necrosis Factor, Alpha-Induced Protein3/ ubiquitin-protein transferase activity and inhibit NF-kappa B activation as well as TNF-mediated apoptosis
Chemokines and Interleukins	CCL2	Chemokine (C-C Motif) Ligand 2/ A member of the CC subfamily contains chemotactic activity for monocytes and basophils
	CXCL1	Chemokine (C-X-C Motif) Ligand 1 (Melanoma Growth Stimulating Activity, Alpha)/ chemotactic activity for neutrophil
	CXCL2	Chemokine (C-X-C Motif) Ligand 2/ produced by activated monocytes and neutrophils and expressed at sites of inflammation
	CXCL6	Chemokine (C-X-C Motif) Ligand 6 /chemotactic activity for neutrophil
	IL-6	Interleukin 6/ cytokine activity and interleukin-6 receptor, relation with B-cells, lymphocyte and monocyte differentiation,inflammation
	IL-8	Chemokine (C-X-C Motif) Ligand 8/ the major mediators of the inflammatory response, chemotactic activity that attracts neutrophils, basophils and T-cell
Immunoreceptors	CD83	CD83 Molecule/ an antigen presentation or the cellular interactions that follow lymphocyte activation
	NOD2	Nucleotide-Binding Oligomerization Domain Containing 2/ protein kinase binding and peptidoglycan binding.
	CFB	Complement Factor B / serine-type endopeptidase activity and complement binding
Cell adhesion molecules and receptors	ICAM1	Intercellular Adhesion Molecule 1/ a cell surface glycoprotein
	NINJ1	Ninjurin 1/ Homophilic cell adhesion molecule
	VCAM1	Vascular Cell Adhesion Molecule 1/ integrin binding and primary amine oxidase activity
Stress response genes	SOD2	Superoxide Dismutase 2, Mitochondrial/ deSTFoys superoxide anions
Enzymes	PTGES	Prostaglandin E Synthase / catalyzes the oxidoreduction of prostaglandin endoperoxide H2 (PGH2) to prostaglandin E2 (PGE2)
Early response genes	TNFAIP2	Tumor Necrosis Factor, Alpha-Induced Protein 2/ a mediator of inflammation and angiogenesis
Miscellaneous	TFPI2	Tissue Factor Pathway Inhibitor 2/ serine-type endopeptidase inhibitor activity and extracellular matrix STFuctural constituent

**Fig. 5:**
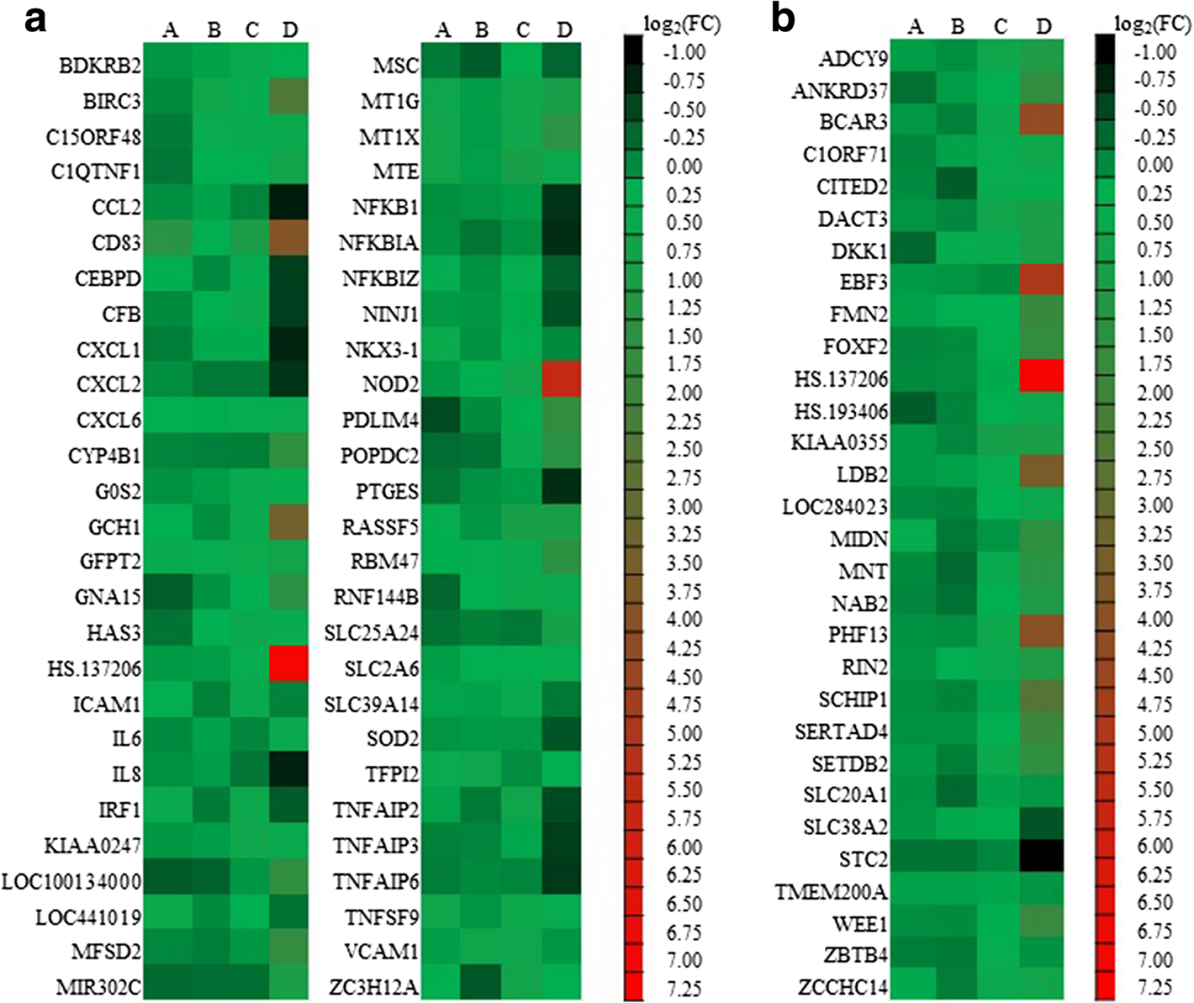
The heat map of target genes of 54 up-regulated genes (**a**) and 30 down-regulated genes (**b**) showed differential pattern of gene expressions in NHDFs pretreated with test compounds prior to IL-1β (1 ng/mL) challenge. Column details: A: indomethacin (50 μM) + IL-1β; B: GA 3 μg/mL+ IL-1β; C: PP 30 μg/mL+ IL-1β and D: STF 3 μg/mL+ IL-1β

The 84 genes which were significantly altered (*P* < 0.05, fold change ≥2 or ≤ 0.5) were considered to be the target genes. To determine the effects of test compounds on IL-1β-treated cells, the expression profiling of 84 genes modulated by STF extracts, GA, PP and indomethacin were observed compared to IL-1β -treated cells, as shown in Fig. 5. In upregulated genes induced by IL-1β, treatment with STF (3 μg/mL) provided greater effects on downregulation of inflammatory and immune genes involved in NF-κB components (NFKB1,NFKBIA,NFKBIZ), chemokines (CCL2,CXCL1,CXCL2,IL8), transcriptional regulator (IRF1), prostaglandin E synthase (PTGES) and TNF alpha induced protein family (TNFAIP2, TNFAIP3, TNFAIP6) than indomethacin in IL-1β-treated cells. Furthermore, STF noticeably induced several gene expressions associated with breast cancer anti-estrogen resistance 3 (BCAR3), cluster of differentiation 83 (CD83), nucleotide-binding oligomerization domain containing 2 (NOD2). Meanwhile, gallic acid (3 μg/mL) appeared to give the greatest inhibition of IL-1β-induced MSC and ZC3H12A genes (Fig. 5a). Additionally, STF reversed the effects of IL-1β-mediated downregulation of some genes such as early B-cell factor 3 (EBF3), LIM domain binding 2 (LDB2) and PHD finger protein 13 (PHF13). Moreover, STF enhanced the effects of IL-1β-mediated downregulation of SLC38A2 and STC genes (Fig. 5b). The overall of gene expression profile modulated by IL-1β, indomethacin, STF and its active compounds were demonstrated in Additional file 4: Table S1.

**Fig. 6:**
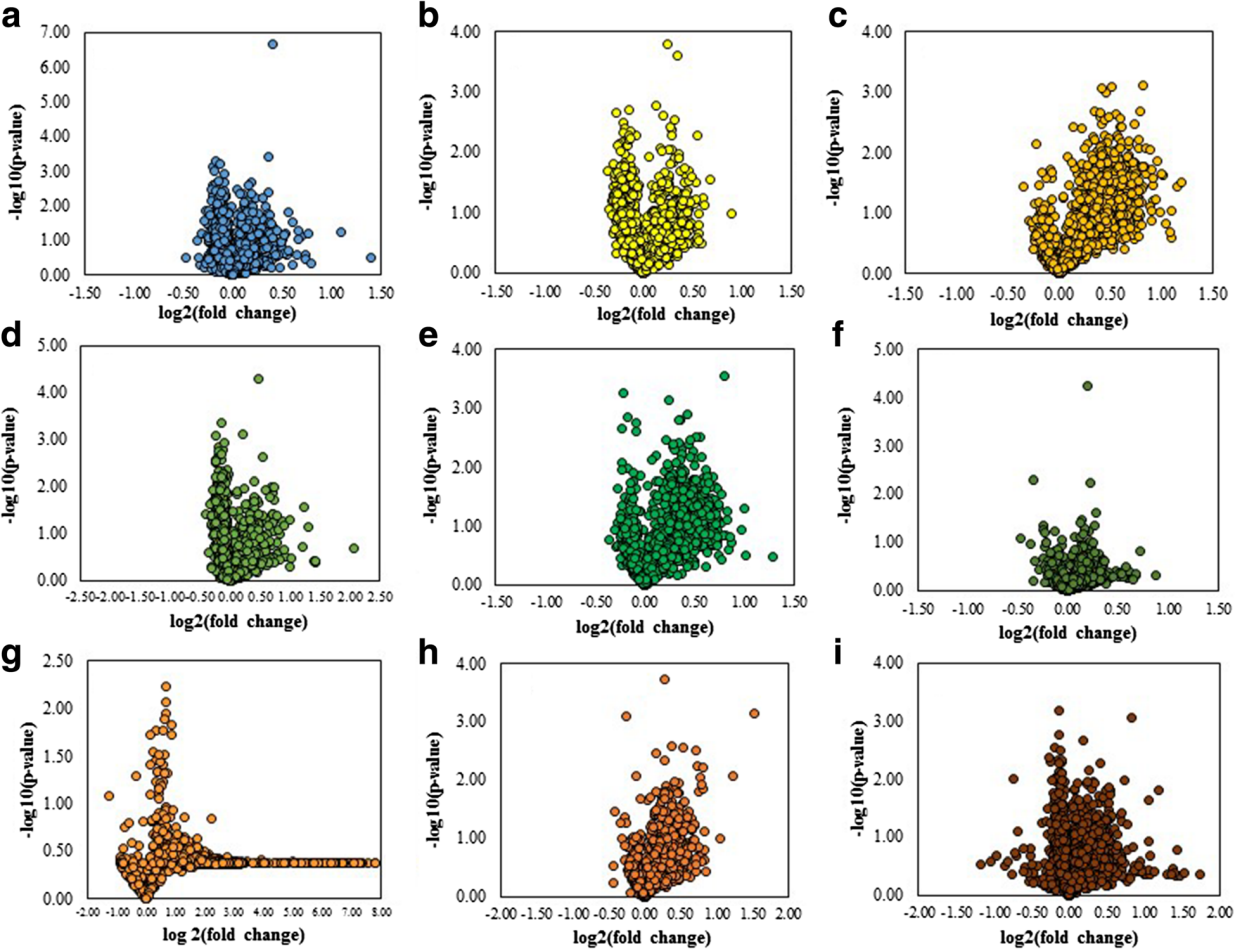
The difference of target gene expression patterns of test compounds in NHDFs induced by IL-1β 1 ng/mL for 4 h (**a-i**); (**a**) Indomethacin 50 μM,(**b**) PP 1 μg/mL,(**c**) PP 30 μg/mL, (**d**) GA 0.3 μg/mL, (**e**) GA 1 μg/mL and (**f**) GA 3 μg/mL, (**g**) STF 3 μg/mL, (**h**) STF 10 μg/mL, **(i**) STF 30 μg/mL

Furthermore, Fig. 6 demonstrated the effect of all test compounds on gene expression pattern following the findings showing 1,653 significantly altered genes in IL-1β-treated cells compared to untreated control cells (Fig. 4a). Treatment with indomethacin (Fig. 6a), PP (Fig. 6b and c), GA (Fig. 6d-f) and STF differently modulated gene expression patterns (Fig. 6g -i).

## Discussion

In this study, we first investigated biologically active compounds of STF using LC separation and its pharmacological effects related to the anti-inflammatory and antioxidant actions using primary human fibroblast culture model and microarray analysis. The HPLC analysis revealed that PP and phenolic compounds including GA were present in STF. In a previous study using reverse-phase HPLC [12], PP was also present in long pepper (*Piper retrofractum* Vahl.) and black pepper (*Piper nigrum* L.). In addition, other previously reported study cited GA and PP as having anti-inflammatory and antioxidant activities and thus both compounds were chosen as reference markers for investigating the effects of STF extracts in our study [13–15].

Since ROS play a role in initiation and progression of inflammatory disorders. Our results indicated that STF and its components were able to scavenge DPPH free radicals, although their abilities to scavenge DPPH were less than ascorbic acid base on IC_30_ values. Among all STF’s components, *Terminalia chebula Retz.* (gall and fruit), showed the strongest free radical scavenging activities. Previous studies also reported that tannins and phenolic compounds, including GA, punicalagin, chebulagic acid, casuarinin and chebulanin found in *Terminalia chebula* Retz. were accountable for its antioxidant potentials [16]. Concurrently, PP standard failed to yield free radical scavenging activities while black pepper and long pepper, which were the major constituents in STF extracts, had minimal ability to scavenge the DPPH radicals. It is possible that free radical scavenging actions of black pepper and long pepper may be attributed to other phytochemical substances [17]. Thus, the DPPH results suggested that GA and other unidentified compounds present in the STF’s components may be responsible for the free radical scavenging activity of the formula.

Inflammatory and oxidative stress events are associated with the production of pro-inflammatory cytokines through NADPH oxidase complex [18] which subsequently produce ROS such as superoxide and hydrogen peroxide in human fibroblasts. The flow cytometry analysis supported that pro-inflammatory cytokine IL-1β in NHDFs can induce ROS formation. Furthermore, this study also explored antioxidant effects of STF extracts, GA and PP on IL-1β-treated NHDFs. Our study demonstrated that pretreatment of the cells with STF and its active compounds can suppress IL-1β-induced intracellular ROS levels. Previous reports also demonstrated that GA and PP showed abilities to promote mRNA levels and activities of antioxidant enzymes and inhibition of lipid peroxidation [14, 19]. Additionally, the concentrations at which STF demonstrated its inhibitory effects on ROS formation were much higher than those of GA. Other unidentified substances in the formula may also contribute to antioxidant actions of the STF extracts.

IL-1β plays critical roles in regulating inflammatory and antioxidant genes related to cytokines, chemokines, adhesion molecules, immunomodulators, apoptotic regulators, and growth factors involved in physiological, pathological and oxidative stress events. In our study, total 84 genes were significantly altered; 54 genes were up-regulated and 30 genes were down regulated in IL-1β-treated NHDFs and, in consistent with previous studies, similar pattern of altered genes or gene expression profiling was observed fibroblast model of inflammation [20, 21]. In addition, it is well known that IL-1 can activate NF-кB in various cell types through canonical NF-кB-activating cascades [22, 23]. Our results indicated that the member of NF-кB transcription components, NFKB1, NFKBIA (IκBα) and NFKBIZ genes, were significantly upregulated in IL-1β-treated NDHFs. Additionally, treatment with IL-1β upregulated the mRNA of SOD2, which serves as the primary antioxidant defense in mitochondria that changes superoxide molecules into hydrogen peroxide and oxygen and could be regulated by NF-кB [24, 25]. The microarray results suggesting altered expression of genes involved in mitochondrial dysfunction were in consistent with the flow cytometric findings showing that the presence of IL-1β enhanced ROS production in NHDFs as augmented ROS generation can be associated with mitochondrial injury [26]. Thus, it is possible that upregulation of antioxidant defenses may reflect an adaptive mechanisms in response to inflammatory challenge.

Currently, it has been suggested that more than 150 target genes including transcription factors, cytokines, chemokines and immune mediators can be regulated by NF-кB [27]. The crosstalk of inflammatory and immunomodulatory pathways with multiple other pathways unrelated to NF-кB signaling is therefore possible [28]. Interestingly, it is well recognized that NOD2 is involved in host defense against bacterial infections and various inflammatory diseases. Previous studies showed that pro-inflammatory cytokines such as IFN-γ, TNF-α are able to upregulate NOD mRNA and protein levels [29]. Furthermore, NOD2 signaling pathway also participates in NF-кB activation [30] resulting in active IL-1β secretion [31]. Additionally, BIRC3 encodes a member of the inhibitor of apoptosis protein (IAP) family, which plays the potential role in caspase-1 modulation in association with inflammasome activation, which could be mediated by IL-1β [32]. Furthermore, IAP encoded by BIRC3 gene is suggested to serve as a regulator of NF-кB signaling [33]. Our study showing upregulation of NOD2 and BIRC3 gene by IL-1β in human fibroblasts supports the role of IL-1β in acute inflammation; moreover, it also contributes to immune activation in fibroblasts through secreting multiple biochemical mediators such as cytokines, chemokines, transcription factors, proteases, and apoptosis regulators involved in the downstream of NF-кB activation.

Interestingly, PANTHER pathway analysis indicated that IL-1β could upregulate interleukin and CCKR signaling in NHDFs (Table 3). Previous evidence showed that IL-1β had the potential to upregulate important genes including NFKBIA and IL-8 involved in activation of NF-кB cascade associated with CCKR signaling [34, 35], as well as IL-6 and IL-8, the critical pro-inflammatory mediators, involved in interleukin signaling. Our findings also indicated the effects of IL-1β on CCKR and prostaglandins (Additional file 3: Table 1) postulated to be involved in pain sensitization [36]. In addition, increased expression of IL-6 and IL-8 genes by IL-1β in our study support the inflammatory role of IL-1β as previous in vitro study observing enhanced IL-6 and IL-8 levels in rheumatoid arthritis synovial fibroblasts [37].

**Table 3:** List of significantly up-regulated genes in IL-1β-treated NHDFs using PANTHER pathway enrichment analysis. *P* < 0.05 was considered statistically significant

No.	Pathway (PANTHER pathway ID)	Gene Symbol	Protein class	*p*-value
1	CCKR signaling map	BIRC3	protease inhibitor	2.17E-02
		CXCL1	chemokine	
		CXCL2	chemokine	
		NFKBIA	phospholipase	
		IL-8	chemokine	
2	Interleukin signaling pathway	IL-6	interleukin superfamily	3.78E-02
		IL-8	chemokine	

We also investigated the effects of indomethacin used as the positive control, STF extracts and its active compounds (GA and PP) on 84 target genes induced by IL-1β (Fig. 5a and b). All test compounds can downregulate the expression of genes involved in NF-кB signaling pathway through three NF-кB modulated pathways [38]. NFKB1 and NFKB1A genes which were significantly down-regulated more than 2-fold by STF (Fig. 5a) participate in the pathways that can counteract the NF-кB upstream signaling including the inhibition of phosphorylation of IKK complexes and IKB degradation, the inhibition of mobilization of NF-кB into cell nucleus and binding with specific DNA sequence.

In addition, our study showed that STF extracts led to a significant down-regulation (>1.5-fold) of IL-1β-induced IRF1 gene involved in COX-2 expression [39]. Moreover, the STF extracts also attenuated many inflammatory chemokine and interleukin genes (Fig. 5a) such as CXCL1, CXCL2, CCL2 IL-6 and IL-8 enhanced by IL-1β which were previously suggested to associate with clinical analgesia [40] and inflammatory hypernociception [41]. On the contrary, all test compounds except STF significantly downregulated (>2-fold) IL-1β-induced expression of CD83, and NOD2 genes. CD83 is associated with the activation of NF-кB binding and increased level of PGE_2_ [42]. Nevertheless, the mechanism by which STF enhances the overexpression of NOD2 gene involved in innate immune and inflammatory responses [43] needs further investigation. Furthermore, STF reversed the effects of IL-1β on downregulation of various genes (Fig. 5b), such as, EBF3 associated with B lymphocyte differentiation [44], bone and neuronal genesis [45] and tumor suppression [46]. Moreover, STF remarkably downregulated expression of STC2 gene involved in endoplasmic reticulum-stress through PERK pathway in nonalcoholic fatty liver disease [47].

Our results revealed for the first time that STF and its active compounds including PP and GA can modulate various genes involved in intracellular signaling cascades including inflammatory and immunomodulatory signaling pathways. However, our work provided only the evidence-based data on gene expression profiles affected by STF using primary fibroblast model of inflammation. Thus, further studies are warranted to elucidate their therapeutic targets integrating diverse signaling pathways and other pharmacological fields such as pharmacokinetics, pharmacodynamics and herb-drug interactions relating with therapeutic efficacy for development of STF as a novel anti-inflammatory agent.

## Conclusions

In summary, HPLC analysis has revealed the presence of GA as the major phenolic in STF extracts and the presence of PP as the major compound in pepper, the main component herb in STF, indicating that both GA and PP could be possible active ingredients in STF extracts. Pathway enrichment analysis of microarray data indicated that IL-1β can enhance various genes involved in interleukin and CCRK signaling pathways in NHDFs. In targeted gene analysis, all test compounds (indomethacin, STF, GA and PP) provided diverse gene expression profiling involving multiple transcriptional factors including NF-кB as well as inflammatory signaling in IL-1β-pretreated NHDFs.

## Additional files

Additional file 1: The overview of microarray analysis work flow (**Figure S1**) and the list of human housekeeping genes matching with gene array in microarray BeadChip (**Table S1**). (DOCX 32 kb)
Additional file 2: The list of primer sequences for verify microarray data (**Table S1**). The qRT-PCR confirmations the effects of IL-1β (**Figure S1**) and test compounds (**Figure S2**) on NHDFs gene expression from microarray experiment. (DOCX 1228 kb)
Additional file 3: The list of significant gene alterations; up-regulation (**Table S1**) and down-regulation (**Table S2**) (DOCX 26 kb)
Additional file 4: The overall of differently targeted gene expression (84 genes) modulated by IL-1β and test compound treatments compared to IL-β alone. (DOCX 25 kb)

## Abbreviations

GO: Gene Ontology
HPLC: high performance liquid chromatography
NF-кB: Nuclear factor kappa-light-chain-enhancer of activated B cells
NHDFs: Primary human dermal fibroblasts
ROS: Reactive oxygen species
STF: Thai herbal Sahatsatara formula

## References

[1] McBeth J, Jones K (2007). Epidemiology of chronic musculoskeletal pain. Best Pract Res Clin Rheumatol.

[2] Supinski GS, Callahan LA (2007). Free radical-mediated skeletal muscle dysfunction in inflammatory conditions. J Appl Physiol.

[3] Zhang JM, An J (2007). Cytokines, inflammation, and pain. Int Anesthesiol Clin.

[4] Daheshia M, Yao JQ (2008). The interleukin 1beta pathway in the pathogenesis of osteoarthritis. J Rheumatol.

[5] Duncan MR, Berman B (1989). Differential regulation of collagen, glycosaminoglycan, fibronectin, and collagenase activity production in cultured human adult dermal fibroblasts by interleukin 1-alpha and beta and tumor necrosis factor-alpha and beta. J Invest Dermatol.

[6] Conaghan PG (2012). A turbulent decade for NSAIDs: update on current concepts of classification, epidemiology, comparative efficacy, and toxicity. Rheumatol Int.

[7] Kakatum N, Jaiarree N, Makchucit S, Itharat A (2012). Antioxidant and anti-inflammatory activities of Thai medicinal plants in Sahasthara remedy for muscle pain treatment. J Med Assoc Thai.

[8] Nootim P, Bunchuailua W, Kapol N (2013). Comparative Efficacy of Sahasthara Capsule VS Diclofenac Tablet for the Relief of Muscle Pain. J Thai Tradit Altern Med.

[9] Pinsornsak P, Kanokkangsadal P, Itharat A (2015). The Clinical Efficacy and Safety of the Sahastara Remedy versus Diclofenac in the Treatment of Osteoarthritis of the Knee: A Double-Blind, Randomized, and Controlled Trial. Evid Based Complement Alternat Med.

[10] Thangboonjit W, Pluemsamran T, Panich U (2014). Comparative Evaluation of Antityrosinase and Antioxidant Activities of Dietary Phenolics and their Activities in Melanoma Cells Exposed to UVA. Siriraj Med J.

[11] Mi H, Muruganujan A, Casagrande JT, Thomas PD (2013). Large-scale gene function analysis with the PANTHER classification system. Nat Protocols.

[12] Kakatum N (2011). Anti-inflammatory Activity of Thai Traditional Remedy Extract for Muscle Pain Treatment Called Sahasthara and Its Plant Ingredients: Faculty of Medicine, Thammasat University.

[13] Yoon CH, Chung SJ, Lee SW, Park YB, Lee SK, Park MC (2013). Gallic acid, a natural polyphenolic acid, induces apoptosis and inhibits proinflammatory gene expressions in rheumatoid arthritis fibroblast-like synoviocytes. Joint Bone Spine.

[14] Mittal R, Gupta RL (2000). In vitro antioxidant activity of piperine. Methods Find Exp Clin Pharmacol.

[15] Bang JS, da Oh H, Choi HM, Sur BJ, Lim SJ, Kim JY, Yang HI, Yoo MC, Hahm DH, Kim KS (2009). Anti-inflammatory and antiarthritic effects of piperine in human interleukin 1beta-stimulated fibroblast-like synoviocytes and in rat arthritis models. Arthritis Res Ther.

[16] Manosroi A, Jantrawut P, Akazawa H, Akihisa T, Manosroi J (2010). Biological activities of phenolic compounds isolated from galls of Terminalia chebula Retz. (Combretaceae). Nat Prod Res.

[17] Nahak G, Sahu R (2011). Phytochemical Evaluation and Antioxidant activity of Piper cubeba and Piper nigrum. J Appl Pharm Sci.

[18] Meier B, Radeke HH, Selle S, Younes M, Sies H, Resch K, Habermehl GG (1989). Human fibroblasts release reactive oxygen species in response to interleukin-1 or tumour necrosis factor-alpha. Biochem J.

[19] Panich U, Onkoksoong T, Limsaengurai S, Akarasereenont P, Wongkajornsilp A (2012). UVA-induced melanogenesis and modulation of glutathione redox system in different melanoma cell lines: the protective effect of gallic acid. J Photochem Photobiol B.

[20] Schramm F, Kern A, Barthel C, Nadaud S, Meyer N, Jaulhac B, Boulanger N (2012). Microarray Analyses of Inflammation Response of Human Dermal Fibroblasts to Different Strains of Borrelia burgdorferi Sensu Stricto. PLoS One.

[21] Vardar-Sengul S, Arora S, Baylas H, Mercola D (2009). Expression Profile of Human Gingival Fibroblasts Induced by Interleukin-1β Reveals Central Role of Nuclear Factor-Kappa B in Stabilizing Human Gingival Fibroblasts During Inflammation. J Periodontol.

[22] Oeckinghaus A, Ghosh S (2009). The NF-κB Family of Transcription Factors and Its Regulation. Cold Spring Harb Perspect Biol.

[23] Karin M, Greten FR (2005). NF-[kappa]B: linking inflammation and immunity to cancer development and progression. Nat Rev Immunol.

[24] Li C, Zhou H-M (2011). The role of manganese superoxide dismutase in inflammation defense. Enzyme Res.

[25] Miao L, St Clair DK (2009). Regulation of superoxide dismutase genes: implications in disease. Free Radic Biol Med.

[26] Kowaltowski AJ, Vercesi AE (1999). Mitochondrial damage induced by conditions of oxidative stress. Free Radic Biol Med.

[27] Pahl HL (1999). Activators and target genes of Rel/NF-kappaB transcription factors. Oncogene.

[28] Oeckinghaus A, Hayden MS, Ghosh S (2011). Crosstalk in NF-[kappa]B signaling pathways. Nat Immunol.

[29] Rosenstiel P, Fantini M, Brautigam K, Kuhbacher T, Waetzig GH, Seegert D, Schreiber S (2003). TNF-alpha and IFN-gamma regulate the expression of the NOD2 (CARD15) gene in human intestinal epithelial cells. Gastroenterology.

[30] Caruso R, Warner N, Inohara N, Nunez G (2014). NOD1 and NOD2: signaling, host defense, and inflammatory disease. Immunity.

[31] Ferwerda G, Kramer M, de Jong D, Piccini A, Joosten LA, Devesaginer I, Girardin SE, Adema GJ, van der Meer JW, Kullberg BJ (2008). Engagement of NOD2 has a dual effect on proIL-1beta mRNA transcription and secretion of bioactive IL-1beta. Eur J Immunol.

[32] Labbé K, McIntire Christian R, Doiron K, Leblanc Philippe M, Saleh M (2011). Cellular Inhibitors of Apoptosis Proteins cIAP1 and cIAP2 Are Required for Efficient Caspase-1 Activation by the Inflammasome. Immunity.

[33] Gyrd-Hansen M, Meier P (2010). IAPs: from caspase inhibitors to modulators of NF-kappaB, inflammation and cancer. Nat Rev Cancer.

[34] Tripathi S, Flobak Å, Chawla K, Baudot A, Doni JN, Skjøndal-Bar N, Bruland T, Thommesen L, Kuiper M, Lægreid A (2013). The Gastrin and Cholecystokinin Receptors mediated signaling network: A scaffold for data analysis and new hypotheses on regulatory mechanisms.

[35] Kunsch C, Rosen CA (1993). NF-kappa B subunit-specific regulation of the interleukin-8 promoter. Mol Cell Biol.

[36] Shavit Y, Wolf G, Goshen I, Livshits D, Yirmiya R (2005). Interleukin-1 antagonizes morphine analgesia and underlies morphine tolerance. Pain.

[37] Georganas C, Liu H, Perlman H, Hoffmann A, Thimmapaya B, Pope RM (2000). Regulation of IL-6 and IL-8 expression in rheumatoid arthritis synovial fibroblasts: the dominant role for NF-kappa B but not C/EBP beta or c-Jun. J Immunol.

[38] Gilmore TD, Herscovitch M (2006). Inhibitors of NF-[kappa]B signaling: 785 and counting. Oncogene.

[39] Blanco JCG, Contursi C, Salkowski CA, DeWitt DL, Ozato K, Vogel SN (2000). Interferon Regulatory Factor (Irf)-1 and Irf-2 Regulate Interferon γ–Dependent Cyclooxygenase 2 Expression. J Exp Med.

[40] Wang X-M, Hamza M, Wu T-X, Dionne RA (2009). Upregulation of IL-6, IL-8 and CCL2 gene expression after acute inflammation: Correlation to clinical pain. Pain.

[41] Verri WA, Cunha TM, Parada CA, Poole S, Cunha FQ, Ferreira SH (2006). Hypernociceptive role of cytokines and chemokines: targets for analgesic drug development?. Pharmacol Ther.

[42] Chen L, Zhu Y, Zhang G, Gao C, Zhong W, Zhang X (2011). CD83-stimulated monocytes suppress T-cell immune responses through production of prostaglandin E2. Proc Natl Acad Sci U S A.

[43] Fritz JH, Ferrero RL, Philpott DJ, Girardin SE (2006). Nod-like proteins in immunity, inflammation and disease. Nat Immunol.

[44] Hagman J, Belanger C, Travis A, Turck CW, Grosschedl R (1993). Cloning and functional characterization of early B-cell factor, a regulator of lymphocyte-specific gene expression. Genes Dev.

[45] Wang SS, Tsai RYL, Reed RR (1997). The Characterization of the Olf-1/EBF-Like HLH Transcription Factor Family: Implications in Olfactory Gene Regulation and Neuronal Development. J Neurosci.

[46] Zhao LY, Niu Y, Santiago A, Liu J, Albert SH, Robertson KD, Liao D (2006). An EBF3-mediated transcriptional program that induces cell cycle arrest and apoptosis. Cancer Res.

[47] Lake AD, Novak P, Hardwick RN, Flores-Keown B, Zhao F, Klimecki WT, Cherrington NJ (2014). The adaptive endoplasmic reticulum stress response to lipotoxicity in progressive human nonalcoholic fatty liver disease. Toxicol Sci.

