# Supplementary material for: IL-1β-induced modulation of gene expression profile in human dermal fibroblasts: the effects of Thai herbal Sahatsatara formula, piperine and gallic acid possessing antioxidant properties
Source: BMC Complement Altern Med. 2017 Jan 10;17:32. doi: 10.1186/s12906-016-1515-0 (PMC5223377; doi:10.1186/s12906-016-1515-0)
Supplement: Additional file 1: — The overview of microarray analysis work flow (Figure S1) and the list of human housekeeping genes matching with gene array in microarray BeadChip (Table S1). (DOCX 32 kb) [file 12906_2016_1515_MOESM1_ESM.docx]

**Additional file 1**

**Microarray data**

- Image capture
- Signal intensity

**Data preprocessing**

- Data normalization
- Data transformation

**Statistical inference**

- Different gene expression

**Selection of gene data sets**

**Differential gene expression analysis**

- Heat map

**Expression pattern analysis**

-Volcano plot

**Gene Ontology analysis**

**-**Functional annotation

**Gene list analyses**

**Statistical hypothesis testing**

***Targeted gene dataset***

**Figure 1** The overview of microarray analysis work flow.

**Table 1** The list of human housekeeping genes matching with gene array in microarray BeadChip. The twelve housekeeping genes were referred from Qiagen website (http://www.sabiosciences.com/rt_pcr_product/HTML/PAHS-000A.html).

| **No.** | **Gene symbols** | **Definition** | **Accession Number** |
| --- | --- | --- | --- |
| 1 | ACTB | Homo sapiens actin, beta (ACTB), mRNA. | NM_001101 |
| 2 | B2M | Homo sapiens beta-2-microglobulin (B2M), mRNA. | NM_004048 |
| 3 | GAPDH | Homo sapiens glyceraldehyde-3-phosphate dehydrogenase (GAPDH), transcript variant 1, mRNA | NM_002046 |
| 4 | GUSB | Homo sapiens glucuronidase, beta (GUSB), transcript variant 1, mRNA. | NM_000181 |
| 5 | HPRT1 | Homo sapiens hypoxanthine phosphoribosyltransferase1 (HPRT1), mRNA. | NM_000194 |
| 6 | HSP90AB1 | Homo sapiens heat shock protein 90kDa alpha (cytosolic), class B member 1 (HSP90AB1), transcript variant 2, mRNA. | NM_007355 |
| 7 | RPL13A | Homo sapiens ribosomal protein L13a (RPL13A), transcript variant 1,  mRNA. | NM_012423 |
| 8 | RPLP0 | Homo sapiens ribosomal protein, large, P0 (RPLP0), transcript variant 1, mRNA. | NM_001002 |
| 9 | PPIA | Homo sapiens peptidylprolyl isomerase A (cyclophilin A) (PPIA), transcript variant 2, mRNA. | NM_203430.1 |
| 10 | TFRC | Homo sapiens transferrin receptor (TFRC), transcript variant 1, mRNA. | NM_003234 |
| 11 | UBC | Homo sapiens ubiquitin C (UBC), mRNA. | NM_021009 |
| 12 | LOC100008588 | Homo sapiens 18S ribosomal RNA (LOC100008588), non-coding RNA. | NR_003286 |
