# Supplementary material for: IL-1β-induced modulation of gene expression profile in human dermal fibroblasts: the effects of Thai herbal Sahatsatara formula, piperine and gallic acid possessing antioxidant properties
Source: BMC Complement Altern Med. 2017 Jan 10;17:32. doi: 10.1186/s12906-016-1515-0 (PMC5223377; doi:10.1186/s12906-016-1515-0)
Supplement: Additional file 2: — The list of primer sequences for verify microarray data (Table S1). The qRT-PCR confirmations the effects of IL-1β (Figure S1) and test compounds (Figure S2) on NHDFs gene expression from microarray experiment. (DOCX 1228 kb) [file 12906_2016_1515_MOESM2_ESM.docx]

**Additional file 2**

| **Gene** | **Name** | | **Primer sequence**  **(5’🡪 3’)** | | **Product size (bp)** | |
| --- | --- | --- | --- | --- | --- | --- |
| CXCL1 | Homo sapiens chemokine (C-X-C motif) ligand 1 (melanoma growth stimulating activity, alpha) (CXCL1), mRNA | F:AACCGAAGTCATAGCCACACT | | 69 | |  |
|  |  | R:TTAACTATGGGGGATGCAGGA | |  |  |  |
| CXCL2 | Homo sapiens chemokine (C-X-C motif) ligand 2 (CXCL2), mRNA | F:TTGTCTCAACCCCGCATCG | | 68 | |  |
|  |  | R:TGGATTTGCCATTTTTCAGCATC | |  |  |  |
| GAPDH | Homo sapiens glyceraldehyde-3-phosphate dehydrogenase (GAPDH), mRNA | F:GACCACTTTGTCAAGCTCATTTCC | | 150 | |  |
|  |  | R:TGAGGGTCTCTCTCTTCCTCTTGT | |  |  |  |
| NFKBIA | Homo sapiens nuclear factor of kappa light polypeptide gene enhancer in B-cells inhibitor, alpha (NFKBIA), mRNA | F:GAAGTGATCCGCCAGGTGAA  R:CTGCTCACAGGCAAGGTGTA | | 189 | |  |
|  |  |  |  |  | |  |
| IL-6 | Homo sapiens interleukin 6 (interferon, beta 2) (IL6), mRNA | F:TGCAATAACCACCCCTGACC | | 163 | |  |
|  |  | R:GTGCCCATGCTACATTTGCC | |  |  |  |

**Table 1** The list of primer sequences for verification of microarray data using qRT-PCR


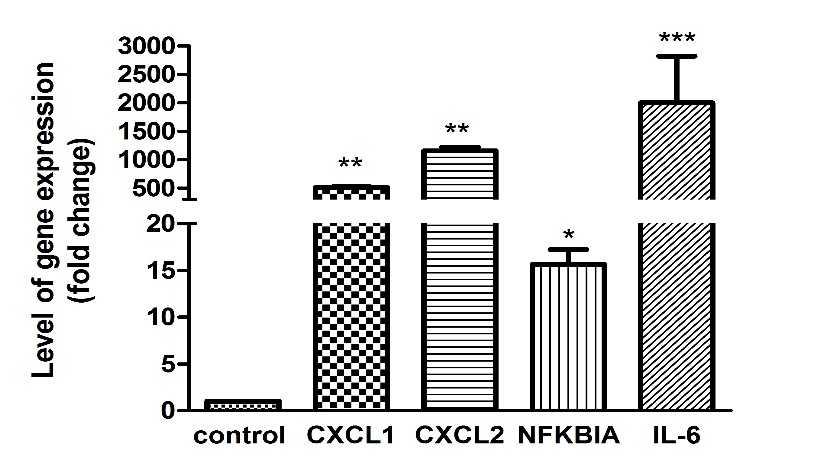

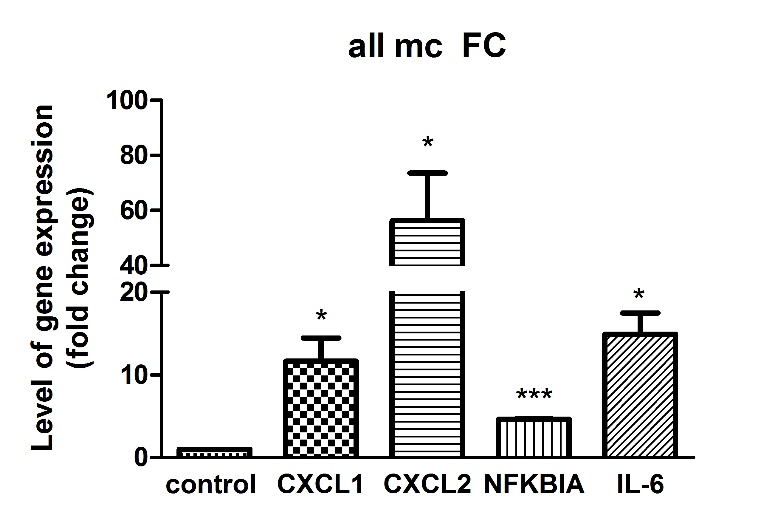


**b**

**a**

**Figure 1** The confirmation of microarray data using qRT-PCR validation. IL-1β has the potential to enhance gene expressions implicating in oxidative stress and inflammation in NHDFs. CXCL1, CXCL2, NFKBIA and IL-6 genes were selected from overall microarray data analysis. The significant differences of microarray analysis **(a)** and qRT-PCR **(b)** were performed using Student’s t-test compared to control group (**P* <0.05; ***P* <0.01; ****P* <0.001) and represented as Mean + SD.

**c**

**d**

**a**

**b**


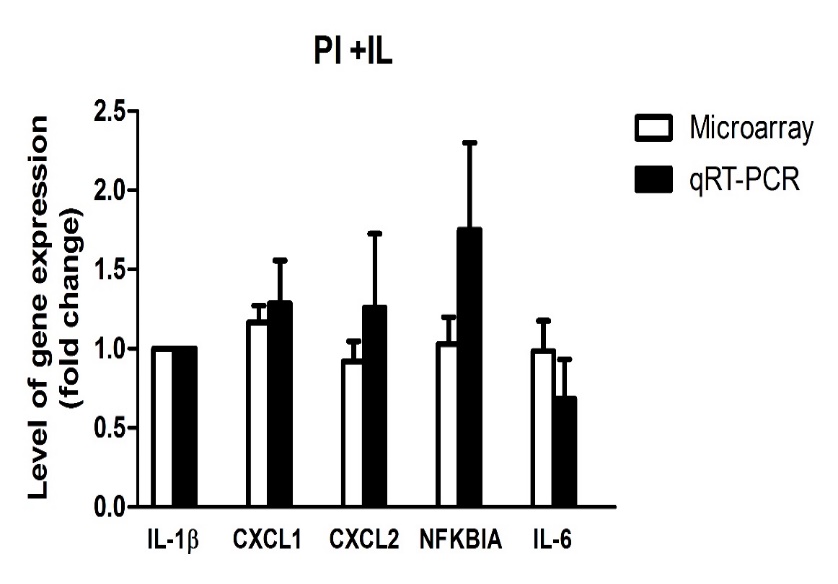

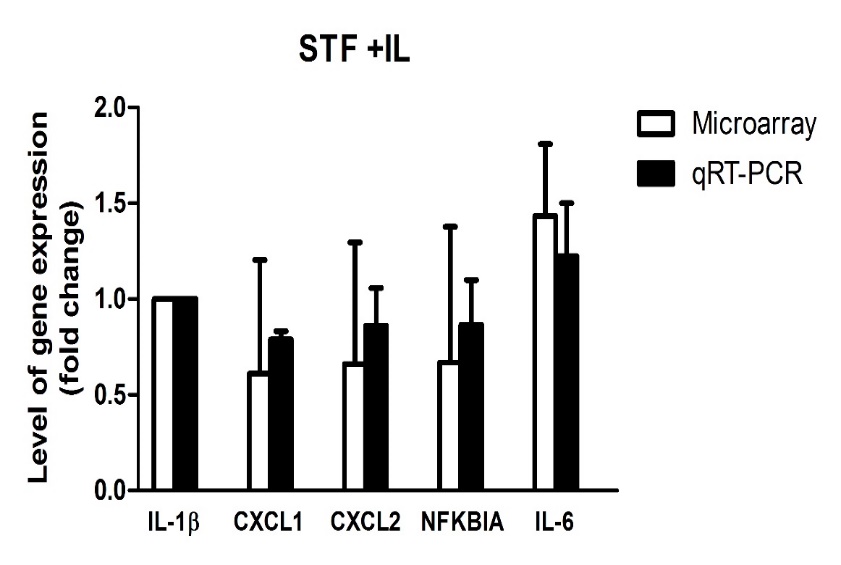

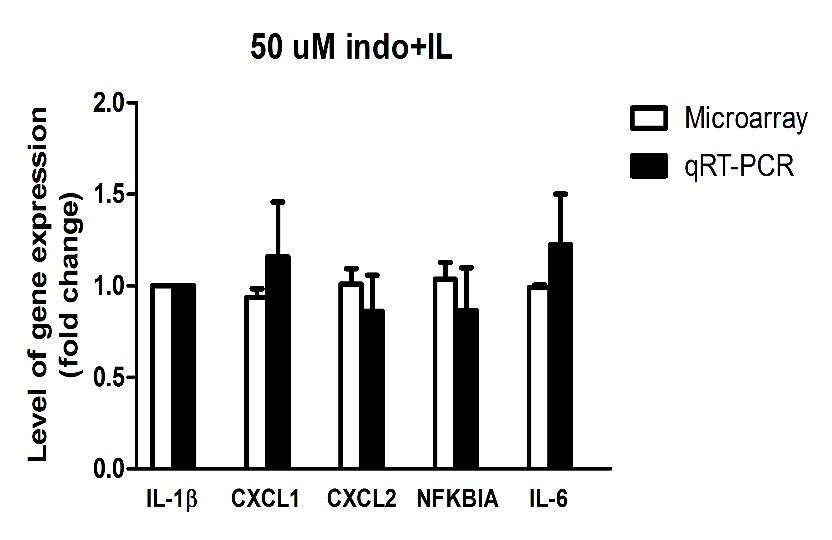

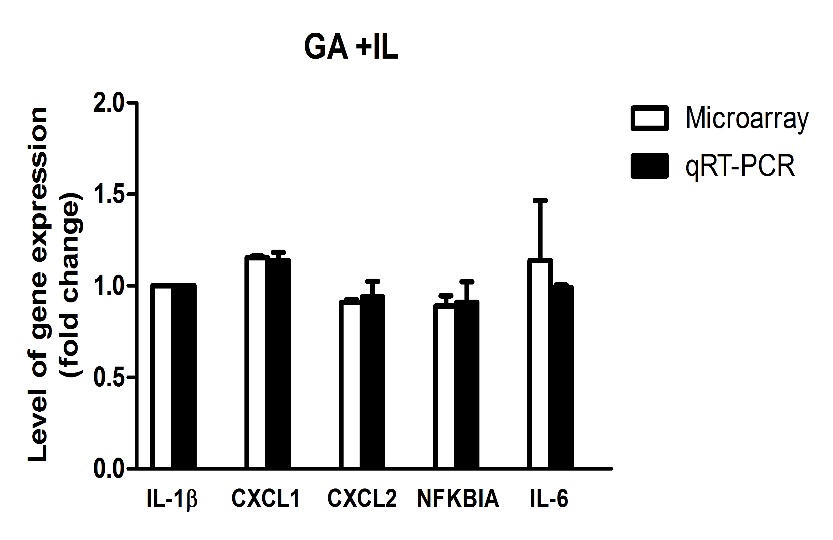


**Figure 2** qRT-PCR confirmation the effects of indomethacin (50 µM) **(a)**, gallic acid (3µg/mL) **(b)**, piperine (30 µg/mL) **(c)** and STF(3µg/mL) **(d)** on gene expression induced by IL-1β. All data were represented as Mean + SD of fold change in triplicate.
