# Supplementary material for: IL-1β-induced modulation of gene expression profile in human dermal fibroblasts: the effects of Thai herbal Sahatsatara formula, piperine and gallic acid possessing antioxidant properties
Source: BMC Complement Altern Med. 2017 Jan 10;17:32. doi: 10.1186/s12906-016-1515-0 (PMC5223377; doi:10.1186/s12906-016-1515-0)
Supplement: Additional file 3: — The list of significant gene alterations; up-regulation (Table S1) and down-regulation (Table S2) (DOCX 26 kb) [file 12906_2016_1515_MOESM3_ESM.docx]

**Additional file 3**

**Table 1** The significant up-regulation (>2-fold) of 54 genes in IL-1β treated groups when compared with untreated groups. Gene definitions and accession numbers were retrieved through NCBI nucleotide database (https://www.ncbi.nlm.nih.gov/nuccore).

| **No.** | **Gene**  **symbol** | **Definition** | **Fold change** | **Accession number** |
| --- | --- | --- | --- | --- |
| 1 | BDKRB2 | Homo sapiens bradykinin receptor B2 (BDKRB2), mRNA. | 3.99 | NM_000623 |
| 2 | BIRC3 | Homo sapiens baculoviral IAP repeat-containing 3 (BIRC3), transcript variant 2, mRNA. | 2.94 | NM_182962.2 |
| 3 | C15ORF48 | Homo sapiens chromosome 15 open reading frame 48 (C15orf48), transcript variant 2, mRNA. | 6.90 | NM_032413.2 |
| 4 | C1QTNF1 | Homo sapiens C1q and tumor necrosis factor related protein 1 (C1QTNF1), mRNA. | 2.67 | NM_198594.1 |
| 5 | CCL2 | Homo sapiens chemokine (C-C motif) ligand 2 (CCL2), mRNA. | 4.36 | NM_002982.3 |
| 6 | CD83 | Homo sapiens CD83 molecule (CD83), transcript variant 1, mRNA. | 3.53 | NM_004233.3 |
| 7 | CEBPD | Homo sapiens CCAAT/enhancer binding protein (C/EBP), delta (CEBPD), mRNA. | 3.57 | NM_005195.2 |
| 8 | CFB | Homo sapiens complement factor B (CFB), mRNA. | 3.97 | NM_001710.4 |
| 9 | CXCL1 | Homo sapiens chemokine (C-X-C motif) ligand 1 (melanoma growth stimulating activity, alpha) (CXCL1), mRNA. | 11.27 | NM_001511.1 |
| 10 | CXCL2 | Homo sapiens chemokine (C-X-C motif) ligand 2 (CXCL2), mRNA. | 53.26 | NM_002089.1 |
| 11 | CXCL6 | Homo sapiens chemokine (C-X-C motif) ligand 6 (granulocyte chemotactic protein 2) (CXCL6), mRNA. | 13.06 | NM_002993.2 |
| 12 | CYP4B1 | Homo sapiens cytochrome P450, family 4, subfamily B, polypeptide 1 (CYP4B1), mRNA. | 2.43 | NM_000779.2 |
| 13 | G0S2 | Homo sapiens G0/G1switch 2 (G0S2), mRNA. | 7.00 | NM_015714.2 |
| 14 | GCH1 | Homo sapiens GTP cyclohydrolase 1 (GCH1), transcript variant 1, mRNA. | 3.89 | NM_000161.2 |
| 15 | GFPT2 | Homo sapiens glutamine-fructose-6-phosphate transaminase 2 (GFPT2), mRNA. | 3.08 | NM_005110.1 |
| 16 | GNA15 | Homo sapiens guanine nucleotide binding protein (G protein), alpha 15 (Gq class) (GNA15), mRNA. | 4.16 | NM_002068.1 |
| 17 | HAS3 | Homo sapiens hyaluronan synthase 3 (HAS3), transcript variant 1, mRNA. | 4.66 | NM_005329.2 |
| 18 | HS.575038 | Homo sapiens cDNA: FLJ21027 fis, clone CAE07110 | 2.55 | Hs.575038 |
| 19 | ICAM1 | Homo sapiens intercellular adhesion molecule 1 (CD54), human rhinovirus receptor (ICAM1), mRNA. | 14.09 | NM_000201.1 |
| 20 | IL6 | Homo sapiens interleukin 6 (interferon, beta 2) (IL6), mRNA. | 14.84 | NM_000600.1 |
| 21 | IL8 | Homo sapiens interleukin 8 (IL8), mRNA. | 12.82 | NM_000584.2 |
| 22 | IRF1 | Homo sapiens interferon regulatory factor 1 (IRF1), mRNA. | 5.84 | NM_002198.1 |
| 23 | KIAA0247 | Homo sapiens KIAA0247 (KIAA0247), mRNA. | 2.01 | NM_014734.2 |
| 24 | LOC100134000 | PREDICTED: Homo sapiens similar to mannosidase, alpha, class 1C, member 1 (LOC100134000), mRNA. | 2.11 | XM_001721795.1 |
| 25 | LOC441019 | PREDICTED: Homo sapiens hypothetical LOC441019 (LOC441019), mRNA. | 2.34 | XM_498969.2 |
| 26 | MFSD2 | Homo sapiens major facilitator superfamily domain containing 2 (MFSD2), mRNA. | 3.03 | NM_032793.2 |
| 27 | MIR302C | Homo sapiens microRNA 302c (MIR302C), microRNA. | 3.50 | NR_029858.1 |
| 28 | MSC | Homo sapiens musculin (activated B-cell factor-1) (MSC), mRNA. | 4.66 | NM_005098.2 |
| 29 | MT1G | Homo sapiens metallothionein 1G (MT1G), mRNA. | 2.30 | NM_005950.1 |

**Table 1** The significant up-regulation (>2-fold) of 54 genes in IL-1β treated groups when compared with untreated groups. Gene definitions and accession numbers were retrieved through NCBI nucleotide database (https://www.ncbi.nlm.nih.gov/nuccore). (cont.)

| **No.** | **Gene symbol** | **Definition** | **Fold change** | **Accession number** |
| --- | --- | --- | --- | --- |
| 30 | MT1X | Homo sapiens metallothionein 1X (MT1X), mRNA. | 2.94 | NM_005952.2 |
| 31 | MTE | Homo sapiens metallothionein E (MTE), mRNA. | 2.26 | NM_175621.2 |
| 32 | NFKB1 | Homo sapiens nuclear factor of kappa light polypeptide gene enhancer in B-cells 1 (NFKB1), mRNA. | 4.57 | NM_003998.2 |
| 33 | NFKBIA | Homo sapiens nuclear factor of kappa light polypeptide gene enhancer in B-cells inhibitor, alpha (NFKBIA), mRNA. | 4.65 | NM_020529.1 |
| 34 | NFKBIZ | Homo sapiens nuclear factor of kappa light polypeptide gene enhancer in B-cells inhibitor, zeta (NFKBIZ), transcript variant 2, mRNA. | 9.48 | NM_001005474.1 |
| 35 | NINJ1 | Homo sapiens ninjurin 1 (NINJ1), mRNA. | 5.84 | NM_004148.2 |
| 36 | NKX3-1 | Homo sapiens NK3 homeobox 1 (NKX3-1), mRNA. | 7.08 | NM_006167.2 |
| 37 | NOD2 | Homo sapiens nucleotide-binding oligomerization domain containing 2 (NOD2), mRNA. | 2.46 | NM_022162.1 |
| 38 | PDLIM4 | Homo sapiens PDZ and LIM domain 4 (PDLIM4), mRNA. | 2.00 | NM_003687.2 |
| 39 | POPDC2 | Homo sapiens popeye domain containing 2 (POPDC2), mRNA. | 2.14 | NM_022135.2 |
| 40 | PTGES | Homo sapiens prostaglandin E synthase (PTGES), mRNA. | 2.30 | NM_004878.3 |
| 41 | RASSF5 | Homo sapiens Ras association (RalGDS/AF-6) domain family member 5 (RASSF5), transcript variant 2, mRNA. | 2.46 | NM_182664.2 |
| 42 | RBM47 | Homo sapiens RNA binding motif protein 47 (RBM47), transcript variant 1, mRNA. | 2.70 | NM_001098634.1 |
| 43 | RNF144B | Homo sapiens ring finger protein 144B (RNF144B), mRNA. | 6.27 | NM_182757.2 |
| 44 | SLC25A24 | Homo sapiens solute carrier family 25 (mitochondrial carrier; phosphate carrier), member 24 (SLC25A24), nuclear gene encoding mitochondrial protein, transcript variant 1, mRNA. | 3.42 | NM_013386.2 |
| 45 | SLC2A6 | Homo sapiens solute carrier family 2 (facilitated glucose transporter), member 6 (SLC2A6), mRNA. | 2.27 | NM_017585.2 |
| 46 | SLC39A14 | Homo sapiens solute carrier family 39 (zinc transporter), member 14 (SLC39A14), mRNA. | 3.72 | NM_015359.1 |
| 47 | SOD2 | Homo sapiens superoxide dismutase 2, mitochondrial (SOD2), nuclear gene encoding mitochondrial protein, transcript variant 1, mRNA. | 6.09 | NM_000636.2 |
| 48 | TFPI2 | Homo sapiens tissue factor pathway inhibitor 2 (TFPI2), mRNA. | 2.14 | NM_006528.2 |
| 49 | TNFAIP2 | Homo sapiens tumor necrosis factor, alpha-induced protein 2 (TNFAIP2), mRNA. | 13.81 | NM_006291.2 |
| 50 | TNFAIP3 | Homo sapiens tumor necrosis factor, alpha-induced protein 3 (TNFAIP3), mRNA. | 13.03 | NM_006290.2 |
| 51 | TNFAIP6 | Homo sapiens tumor necrosis factor, alpha-induced protein 6 (TNFAIP6), mRNA. | 15.28 | NM_007115.2 |
| 52 | TNFSF9 | Homo sapiens tumor necrosis factor (ligand) superfamily, member 9 (TNFSF9), mRNA. | 3.35 | NM_003811.2 |
| 53 | VCAM1 | Homo sapiens vascular cell adhesion molecule 1 (VCAM1), transcript variant 2, mRNA. | 9.16 | NM_080682.1 |
| 54 | ZC3H12A | Homo sapiens zinc finger CCCH-type containing 12A (ZC3H12A), mRNA. | 8.40 | NM_025079.1 |

**Table 2** The significant down-regulation (< 0.5-fold) of 30 genes in IL-1β treated groups when compared with non-treated groups. Gene definitions and accession numbers were retrieved through NCBI nucleotide database (https://www.ncbi.nlm.nih.gov/nuccore).

| **No.** | **Gene symbol** | | **Definition** | | **Fold change** | | **Accession number** |
| --- | --- | --- | --- | --- | --- | --- | --- |
| 1 | ADCY9 | Homo sapiens adenylate cyclase 9 (ADCY9), mRNA. | | 0.47 | | NM_001116.2 | |
| 2 | ANKRD37 | Homo sapiens ankyrin repeat domain 37 (ANKRD37), mRNA. | | 0.25 | | NM_181726.1 | |
| 3 | BCAR3 | Homo sapiens breast cancer anti-estrogen resistance 3 (BCAR3), mRNA. | | 0.46 | | NM_003567.2 | |
| 4 | C1ORF71 | Homo sapiens chromosome 1 open reading frame 71 (C1orf71), mRNA. | | 0.49 | | NM_152609.1 | |
| 5 | CITED2 | Homo sapiens Cbp/p300-interacting transactivator, with Glu/Asp-rich carboxy-terminal domain, 2 (CITED2), transcript variant 1, mRNA. | | 0.30 | | NM_006079.3 | |
| 6 | DACT3 | Homo sapiens dapper, antagonist of beta-catenin, homolog 3 (Xenopus laevis) (DACT3), mRNA. | | 0.47 | | NM_145056.1 | |
| 7 | DKK1 | Homo sapiens dickkopf homolog 1 (Xenopus laevis) (DKK1), mRNA. | | 0.13 | | NM_012242.2 | |
| 8 | EBF3 | Homo sapiens early B-cell factor 3 (EBF3), mRNA. | | 0.48 | | NM_001005463.1 | |
| 9 | FMN2 | Homo sapiens formin 2 (FMN2), mRNA. | | 0.34 | | NM_020066.3 | |
| 10 | FOXF2 | Homo sapiens forkhead box F2 (FOXF2), mRNA. | | 0.34 | | NM_001452.1 | |
| 11 | HS.137206 | Homo sapiens mRNA; cDNA DKFZp564H1663 (from clone DKFZp564H1663) | | 0.45 | | Hs.137206 | |
| 12 | HS.193406 | Homo sapiens cDNA FLJ34755 fis, clone NHNPC1000034 | | 0.36 | | Hs.193406 | |
| 13 | KIAA0355 | Homo sapiens KIAA0355 (KIAA0355), mRNA. | | 0.47 | | NM_014686.2 | |
| 14 | LDB2 | Homo sapiens LIM domain binding 2 (LDB2), mRNA. | | 0.46 | | NM_001290.2 | |
| 15 | LOC284023 | PREDICTED: Homo sapiens hypothetical protein LOC284023, transcript variant 3 (LOC284023), mRNA. | | 0.37 | | XM_933997.1 | |
| 16 | MIDN | Homo sapiens midnolin (MIDN), mRNA. | | 0.41 | | NM_177401.4 | |
| 17 | MNT | Homo sapiens MAX binding protein (MNT), mRNA. | | 0.33 | | NM_020310.2 | |
| 18 | NAB2 | Homo sapiens NGFI-A binding protein 2 (EGR1 binding protein 2) (NAB2), mRNA. | | 0.35 | | NM_005967.2 | |
| 19 | PHF13 | Homo sapiens PHD finger protein 13 (PHF13), mRNA. | | 0.48 | | NM_153812.1 | |
| 20 | RIN2 | Homo sapiens Ras and Rab interactor 2 (RIN2), mRNA. | | 0.36 | | NM_018993.2 | |
| 21 | SCHIP1 | Homo sapiens schwannomin interacting protein 1 (SCHIP1), mRNA. | | 0.44 | | NM_014575.1 | |
| 22 | SERTAD4 | Homo sapiens SERTA domain containing 4 (SERTAD4), mRNA. | | 0.22 | | NM_019605.2 | |
| 23 | SETDB2 | Homo sapiens SET domain, bifurcated 2 (SETDB2), mRNA. | | 0.46 | | NM_031915.1 | |
| 24 | SLC20A1 | Homo sapiens solute carrier family 20 (phosphate transporter), member 1 (SLC20A1), mRNA. | | 0.32 | | NM_005415.3 | |
| 25 | SLC38A2 | Homo sapiens solute carrier family 38, member 2 (SLC38A2), mRNA. | | 0.33 | | NM_018976.3 | |
| 26 | STC2 | Homo sapiens stanniocalcin 2 (STC2), mRNA. | | 0.47 | | NM_003714.2 | |
| 27 | TMEM200A | Homo sapiens transmembrane protein 200A (TMEM200A), mRNA. | | 0.41 | | NM_052913.2 | |
| 28 | WEE1 | Homo sapiens WEE1 homolog (S. pombe) (WEE1), mRNA. | | 0.27 | | NM_003390.2 | |
| 29 | ZBTB4 | Homo sapiens zinc finger and BTB domain containing 4 (ZBTB4), mRNA. | | 0.50 | | NM_020899.2 | |
| 30 | ZCCHC14 | Homo sapiens zinc finger, CCHC domain containing 14 (ZCCHC14), mRNA. | | 0.46 | | NM_015144.1 | |
