# Supplementary material for: IL-1β-induced modulation of gene expression profile in human dermal fibroblasts: the effects of Thai herbal Sahatsatara formula, piperine and gallic acid possessing antioxidant properties
Source: BMC Complement Altern Med. 2017 Jan 10;17:32. doi: 10.1186/s12906-016-1515-0 (PMC5223377; doi:10.1186/s12906-016-1515-0)
Supplement: Additional file 4: — The overall of differently targeted gene expression (84 genes) modulated by IL-1β and test compound treatments compared to IL-β alone. (DOCX 25 kb) [file 12906_2016_1515_MOESM4_ESM.docx]

**Additional file 4**

**Table 1** The overall of differently targeted gene expression (84 genes) modulated by IL-1β and test compound treatments compared to IL-β (1 ng/mL) alone. Column details: A; 50µM indomethacin+ IL-β 1 ng/mL, B; GA 3µg/mL+ IL-β 1 ng/mL, C; PP 30µg/mL+ IL-β 1 ng/mL and D; STF 3µg/mL+ IL-β 1 ng/mL. Abbreviations: U; upregulation, D; down-regulation and ***** compared to control (untreated cells)

| **No.** | **Gene**  **symbol** | **Treatment group** | | | | |
| --- | --- | --- | --- | --- | --- | --- |
|  |  | **IL-1β*** | **A** | **B** | **C** | **D** |
| **1** | **BDKRB2** | **U** | **D** | **D** | **D** | **D** |
| **2** | **BIRC3** | **U** | **D** | **D** | **D** | **U** |
| **3** | **C15ORF48** | **U** | **D** | **D** | **D** | **D** |
| **4** | **C1QTNF1** | **U** | **D** | **D** | **D** | **D** |
| **5** | **CCL2** | **U** | **D** | **D** | **D** | **D** |
| **6** | **CD83** | **U** | **U** | **D** | **U** | **U** |
| **7** | **CEBPD** | **U** | **D** | **D** | **D** | **D** |
| **8** | **CFB** | **U** | **D** | **D** | **D** | **D** |
| **9** | **CXCL1** | **U** | **D** | **D** | **D** | **D** |
| **10** | **CXCL2** | **U** | **D** | **D** | **D** | **D** |
| **11** | **CXCL6** | **U** | **D** | **D** | **D** | **D** |
| **12** | **CYP4B1** | **U** | **D** | **D** | **D** | **U** |
| **13** | **G0S2** | **U** | **D** | **D** | **D** | **D** |
| **14** | **GCH1** | **U** | **D** | **D** | **D** | **U** |
| **15** | **GFPT2** | **U** | **D** | **D** | **D** | **D** |
| **16** | **GNA15** | **U** | **D** | **D** | **D** | **U** |
| **17** | **HAS3** | **U** | **D** | **D** | **D** | **D** |
| **18** | **HS.575038** | **U** | **D** | **D** | **D** | **U** |
| **19** | **ICAM1** | **U** | **D** | **D** | **D** | **D** |
| **20** | **IL6** | **U** | **D** | **D** | **D** | **D** |
| **21** | **IL8** | **U** | **D** | **D** | **D** | **D** |
| **22** | **IRF1** | **U** | **D** | **D** | **D** | **D** |
| **23** | **KIAA0247** | **U** | **D** | **D** | **D** | **D** |
| **24** | **LOC100134000** | **U** | **D** | **D** | **D** | **U** |
| **25** | **LOC441019** | **U** | **D** | **D** | **D** | **D** |
| **26** | **MFSD2** | **U** | **D** | **D** | **D** | **U** |
| **27** | **MIR302C** | **U** | **D** | **D** | **D** | **D** |
| **28** | **MSC** | **U** | **D** | **D** | **D** | **D** |
| **29** | **MT1G** | **U** | **D** | **D** | **D** | **D** |
| **30** | **MT1X** | **U** | **D** | **D** | **D** | **U** |
| **31** | **MTE** | **U** | **D** | **D** | **D** | **D** |
| **32** | **NFKB1** | **U** | **D** | **D** | **D** | **D** |
| **33** | **NFKBIA** | **U** | **D** | **D** | **D** | **D** |
| **34** | **NFKBIZ** | **U** | **D** | **D** | **D** | **D** |
| **35** | **NINJ1** | **U** | **D** | **D** | **D** | **D** |
| **36** | **NKX3-1** | **U** | **D** | **D** | **D** | **D** |
| **37** | **NOD2** | **U** | **D** | **D** | **D** | **U** |
| **38** | **PDLIM4** | **U** | **D** | **D** | **D** | **U** |
| **39** | **POPDC2** | **U** | **D** | **D** | **D** | **U** |
| **40** | **PTGES** | **U** | **D** | **D** | **D** | **D** |

**Table 1** The overall of differently targeted gene expression (84 genes) modulated by IL-1β and test compound treatments compared to IL-β (1 ng/mL) alone. Column details: A; 50µM indomethacin+ IL-β 1 ng/mL, B; GA 3µg/mL+ IL-β 1 ng/mL, C; PP 30µg/mL+ IL-β 1 ng/mL and D; STF 3µg/mL+ IL-β 1 ng/mL. Abbreviations: U; upregulation, D; down-regulation and ***** compared to control (untreated cells) (cont.)

| **No.** | **Gene**  **symbol** | **Treatment group** | | | | |
| --- | --- | --- | --- | --- | --- | --- |
|  |  | **IL-1β*** | **A** | **B** | **C** | **D** |
| **41** | **RASSF5** | **U** | **D** | **D** | **D** | **D** |
| **42** | **RBM47** | **U** | **D** | **D** | **D** | **U** |
| **43** | **RNF144B** | **U** | **D** | **D** | **D** | **D** |
| **44** | **SLC25A24** | **U** | **D** | **D** | **D** | **D** |
| **45** | **SLC2A6** | **U** | **D** | **D** | **D** | **D** |
| **46** | **SLC39A14** | **U** | **D** | **D** | **D** | **D** |
| **47** | **SOD2** | **U** | **D** | **D** | **D** | **D** |
| **48** | **TFPI2** | **U** | **D** | **D** | **D** | **D** |
| **49** | **TNFAIP2** | **U** | **D** | **D** | **D** | **D** |
| **50** | **TNFAIP3** | **U** | **D** | **D** | **D** | **D** |
| **51** | **TNFAIP6** | **U** | **D** | **D** | **D** | **D** |
| **52** | **TNFSF9** | **U** | **D** | **D** | **D** | **D** |
| **53** | **VCAM1** | **U** | **D** | **D** | **D** | **D** |
| **54** | **ZC3H12A** | **U** | **D** | **D** | **D** | **D** |
| **55** | **ADCY9** | **D** | **D** | **D** | **D** | **U** |
| **56** | **ANKRD37** | **D** | **D** | **D** | **D** | **U** |
| **57** | **BCAR3** | **D** | **D** | **D** | **D** | **U** |
| **58** | **C1ORF71** | **D** | **D** | **D** | **D** | **D** |
| **59** | **CITED2** | **D** | **D** | **D** | **D** | **D** |
| **60** | **DACT3** | **D** | **D** | **D** | **D** | **U** |
| **61** | **DKK1** | **D** | **D** | **D** | **D** | **U** |
| **62** | **EBF3** | **D** | **D** | **D** | **D** | **U** |
| **63** | **FMN2** | **D** | **D** | **D** | **D** | **U** |
| **64** | **FOXF2** | **D** | **D** | **D** | **D** | **U** |
| **65** | **HS.137206** | **D** | **D** | **D** | **D** | **U** |
| **66** | **HS.193406** | **D** | **D** | **D** | **D** | **D** |
| **67** | **KIAA0355** | **D** | **D** | **D** | **U** | **U** |
| **68** | **LDB2** | **D** | **D** | **D** | **D** | **U** |
| **69** | **LOC284023** | **D** | **D** | **D** | **D** | **D** |
| **70** | **MIDN** | **D** | **D** | **D** | **D** | **U** |
| **71** | **MNT** | **D** | **D** | **D** | **D** | **U** |
| **72** | **NAB2** | **D** | **D** | **D** | **D** | **U** |
| **73** | **PHF13** | **D** | **D** | **D** | **D** | **U** |
| **74** | **RIN2** | **D** | **D** | **D** | **D** | **U** |
| **75** | **SCHIP1** | **D** | **D** | **D** | **D** | **U** |
| **76** | **SERTAD4** | **D** | **D** | **D** | **D** | **U** |
| **77** | **SETDB2** | **D** | **D** | **D** | **D** | **U** |
| **78** | **SLC20A1** | **D** | **D** | **D** | **D** | **D** |
| **79** | **SLC38A2** | **D** | **D** | **D** | **D** | **D** |
| **80** | **STC2** | **D** | **D** | **D** | **D** | **D** |
| **81** | **TMEM200A** | **D** | **D** | **D** | **D** | **D** |
| **82** | **WEE1** | **D** | **D** | **D** | **D** | **U** |
| **83** | **ZBTB4** | **D** | **D** | **D** | **D** | **D** |
| **84** | **ZCCHC14** | **D** | **D** | **D** | **D** | **D** |
